# Supplementary figures and images for: Large-bodied ornithomimosaurs inhabited Appalachia during the Late Cretaceous of North America
Source: PLoS One. 2022 Oct 19;17(10):e0266648. doi: 10.1371/journal.pone.0266648 (PMC9581415; doi:10.1371/journal.pone.0266648)

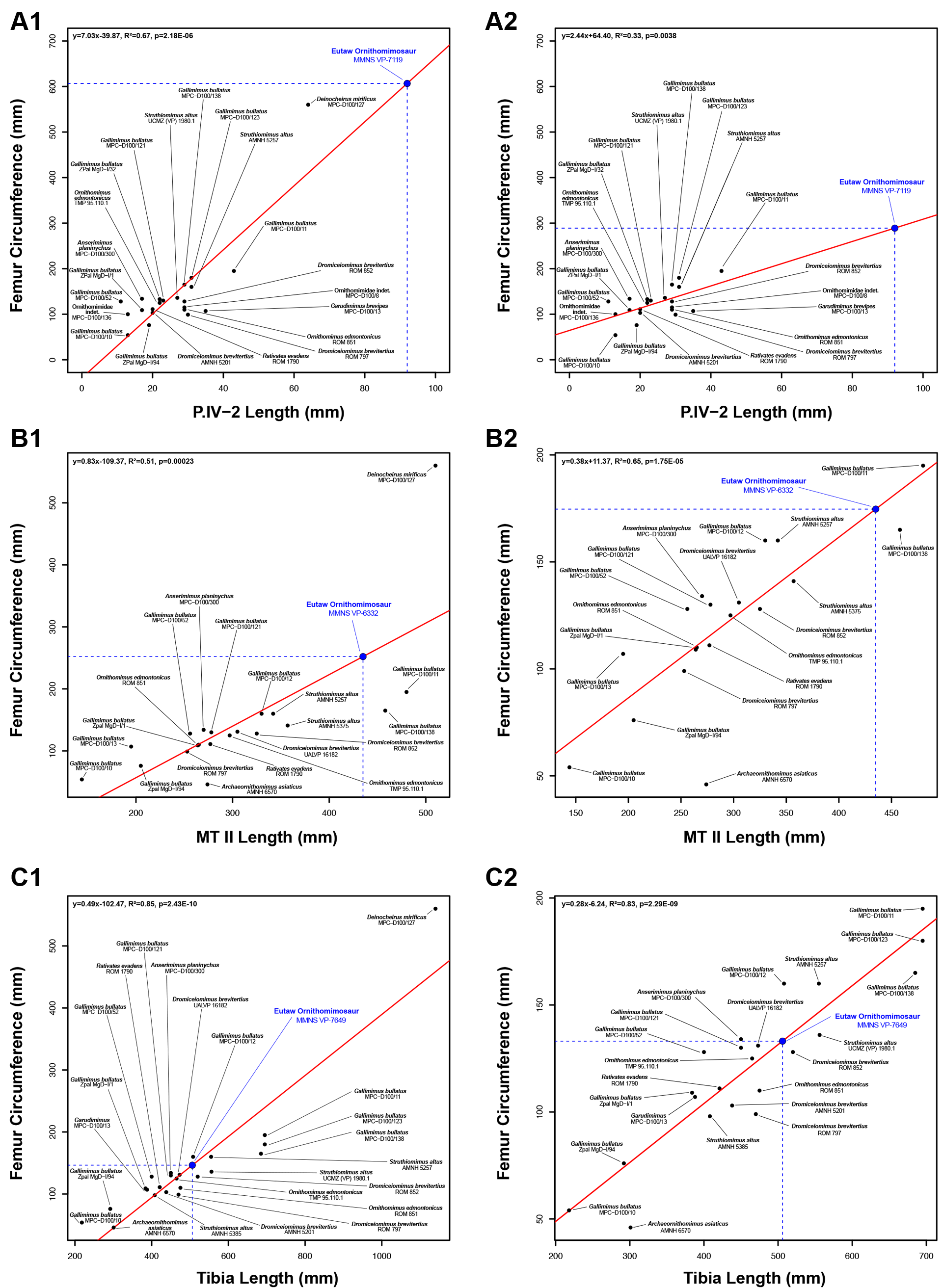

Supplement: S1 Fig — Femoral circumference versus (A1, A2), pedal phalanx IV-2 lengths, (B1, B2), metatarsal II lengths, and (C1, C2) tibial lengths. Note that the graphs of A1, B1, and C1 show when D. mirificus is included in the analysis, and A2, B2, and C2 show when D. mirificus is excluded from the analysis. (TIF) [file pone.0266648.s001.tif]

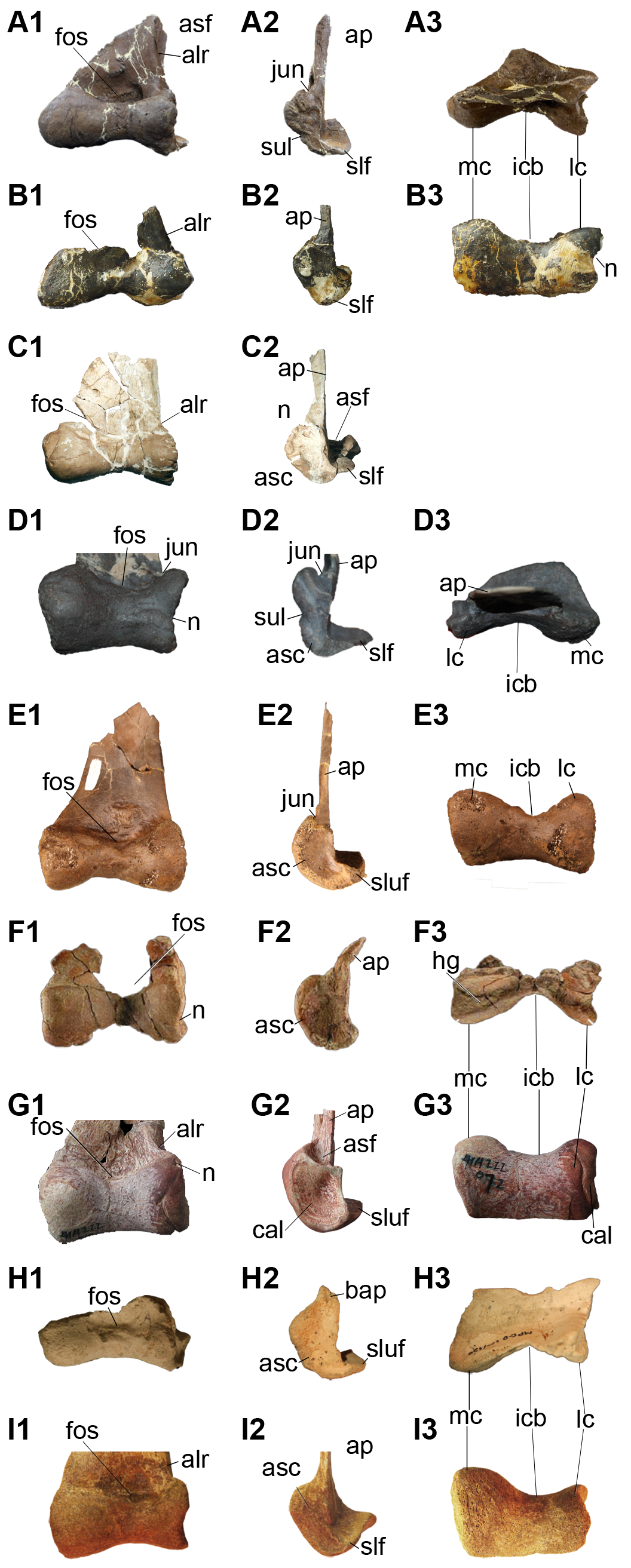

Supplement: S2 Fig — (A), T. rex (MOR 1125); (B), D. aquilunguis (ANSP 9995); (C), A. montgomeriensis (RMM 6670); (D), F. utahensis (UMNH VP 12364); (E), Anzu sp. (NCSM 33801); (F), T. sampsoni (UMNH VP 19479); (G), Q. henanensis (HGM 41HIII-0106); (H), A. tugrikinensis (MPC-D 100/130). Abbreviations: alr, anterolateral ridge; ap, ascending process; asc, articular surface for the calcaneum; asf, articular surface for the fibula; bap, base of the ascending process; cal, calcaneum; fos, median fossa; hg, horizontal groove; icb, intercondylar bridge; jun, junction; lc, lateral condyle; mc, medial condyle; n, notch; slf, laterally flared articular surface of the base; sluf, laterally unflared articular surface of the base. Note that all astragali refer to left except for the right side of F. utahensis and is reversed. (TIF) [file pone.0266648.s002.tif]

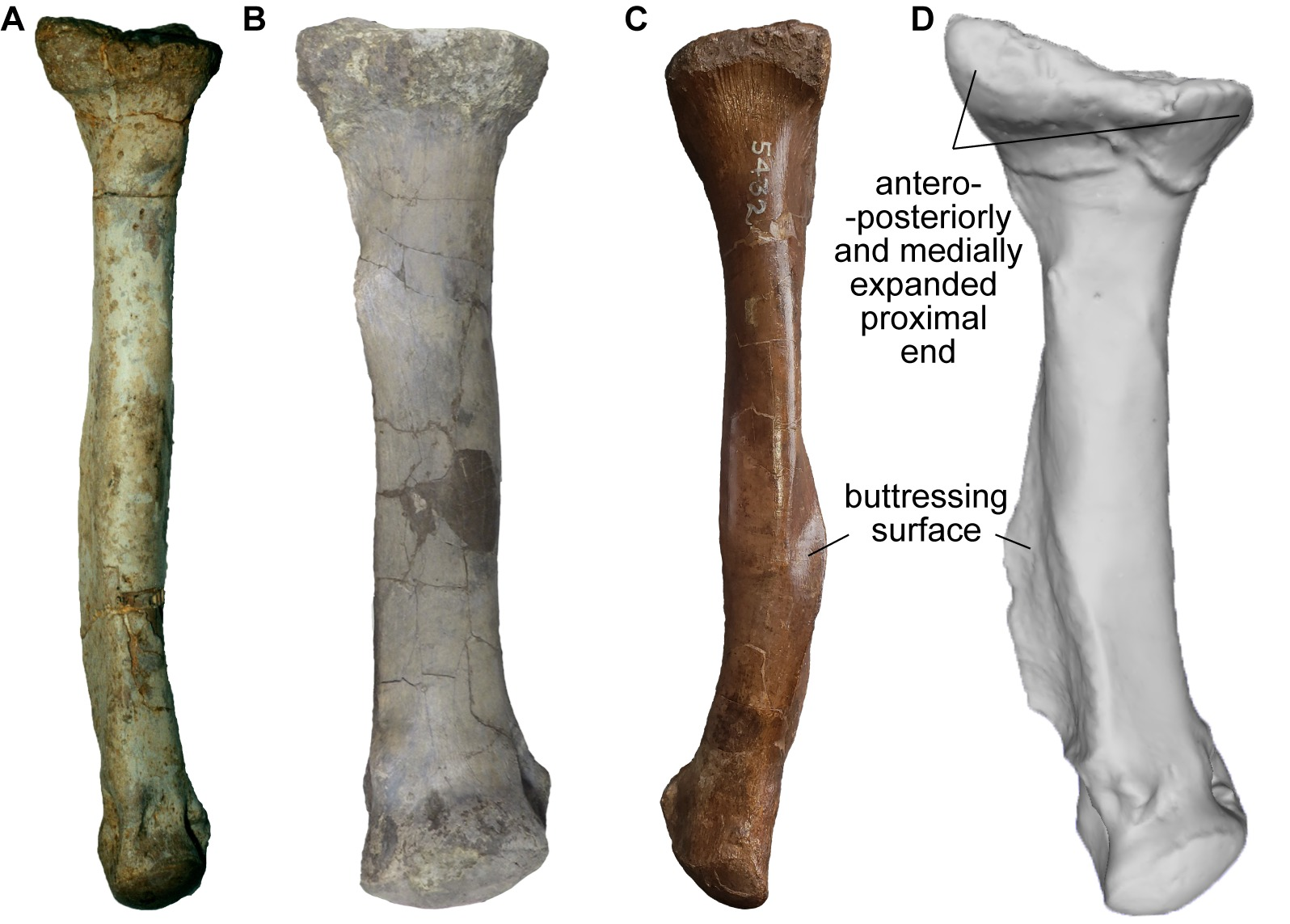

Supplement: S3 Fig — (A), A. montgomeriensis (RMM 6670); (B), A. atokensis (NCSM 14345); (C), A. sarcophagus (AMNH 5432); (D), T. rex (FMNH PR 2081, 3D reconstructed image). Note that all images refer to the right metatarsals except for the left side of A. sarcophagus. All images are not to scale. (TIF) [file pone.0266648.s003.tif]

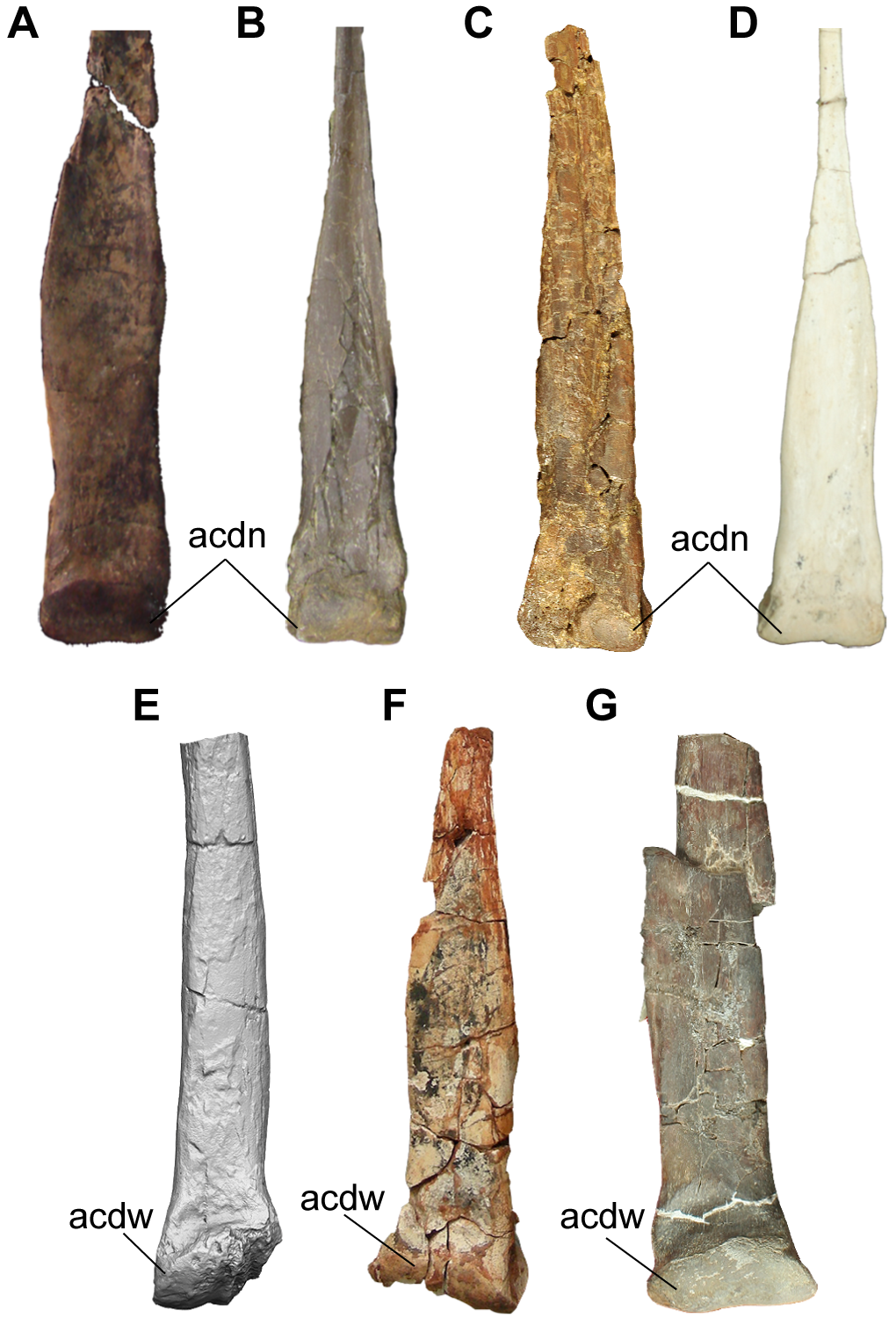

Supplement: S4 Fig — (A), O. velox (YPM 542); (B), D. brevitertius (UA 16182); (C), R. evadens (ROM 1790); (D), A. tugrikinensis (MPC-D 100/130); (E), A. fridayi (UAM 74–16, 3D reconstructed image); (F), Q. henanensis (HGM 41HIII-0106); (G), B. grandis (FRDC-GS GJ 06). Abbreviations: acdn, distally mediolaterally not widened articular caput; acdw, distally mediolaterally widened articular caput. Note that all images refer to left third metatarsals except for the right side of large Gansu ornithomimid and Arkansaurus fridayi. All images are not to scale. (TIF) [file pone.0266648.s004.tif]

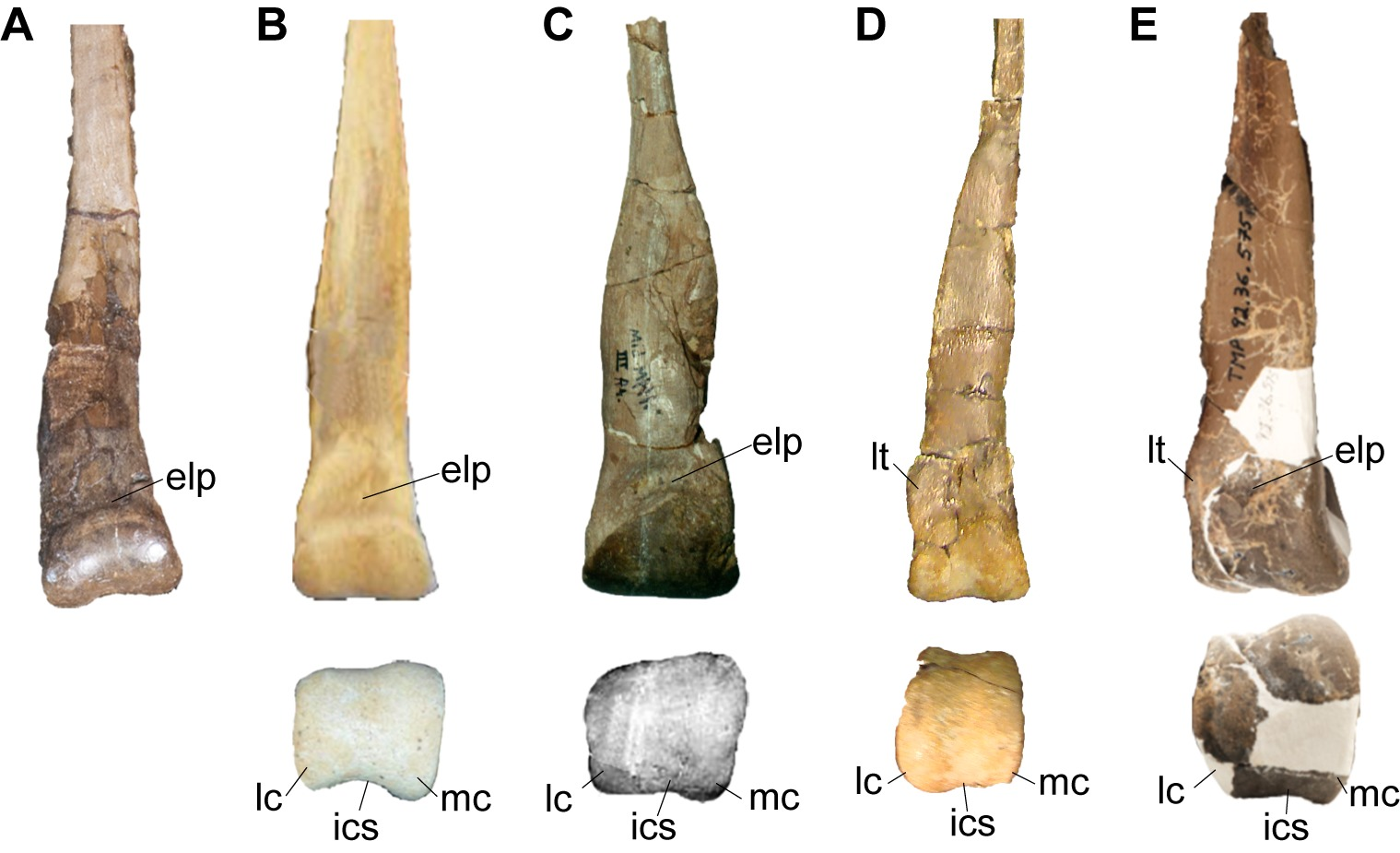

Supplement: S5 Fig — (A), C. pergracilis (= M. canadensis), (CMN 8538); (B), E. rarus (MPC-D 102/6); (C), A. montgomeriensis (RMM 6670); (D), T. sampsoni (UMNH VP 19479); (E), S. inequalis (= L. mcmasterae) (TMP 1992.036.0575). Abbreviations: elp, extensor ligament pit; ics, intercondylar sulcus; lc, lateral condyle; lt, “lateral tab,”; mc, medial condyle. All images are not to scale. (TIF) [file pone.0266648.s005.tif]

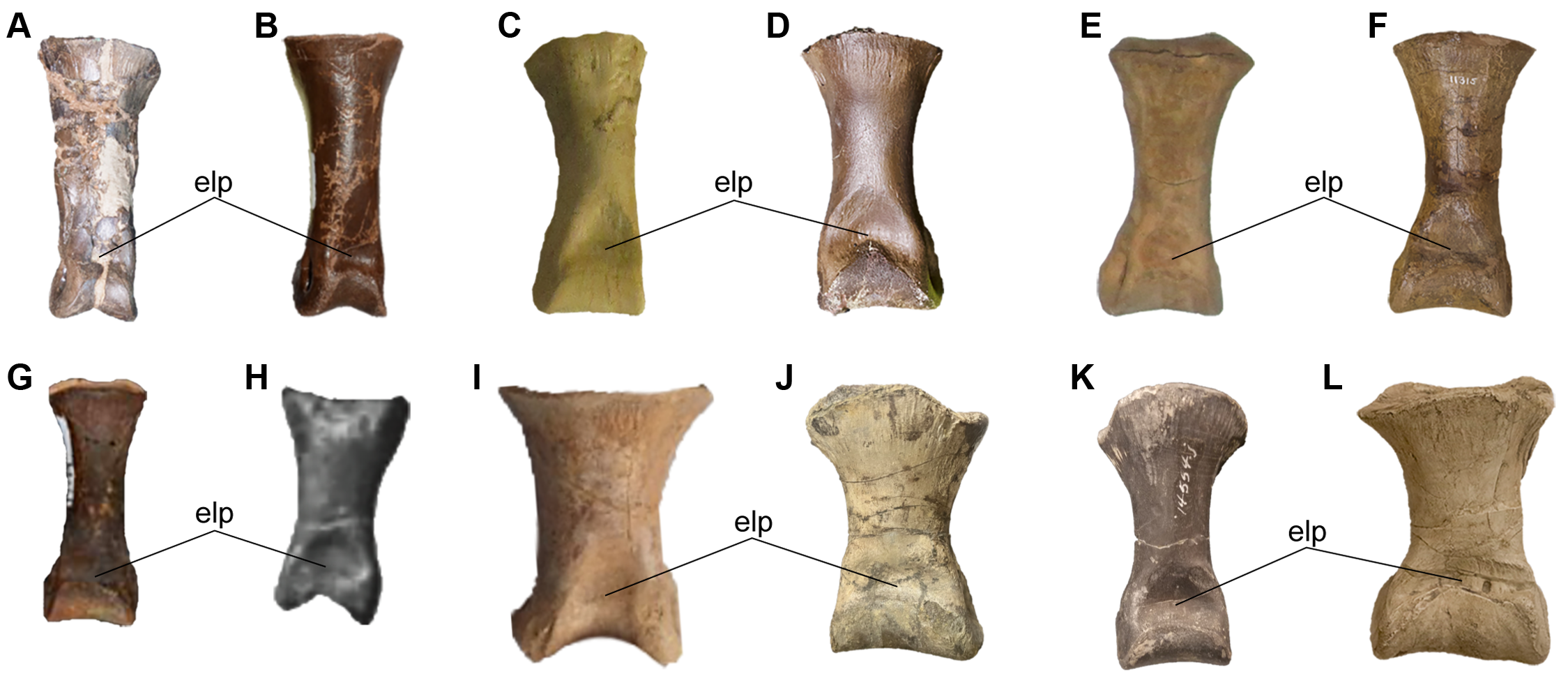

Supplement: S6 Fig — (A), C. pergracilis (= M. canadensis) (CMN 8538); (B), A. wyliei (CMN 78000); (C), juvenile T. bataar (MPC-D 107/7); (D), G. libratus (FMNH PR 2211); (E), Alectrosaurus sp. (MPC-D 100/51); (F), A. sarcophagus (CMN 11315); (G), T. sampsoni (UMNH VP-19479); (H), F. utahensis (UMNH VP 12362); (I), G. erlianensis (LH V0011); (J), A. atokensis (NCSM 14345); (K), A. fragilis (YPM 4944); (L), T. rex (FMNH PR 2081). Abbreviations: elp, extensor ligament pit. Note that A, D, F, J, and L are referred to the right and B, C, E, G-I, and K are referred to the left pedal phalanges. Note that A and L are mirrored, and all images are not to scale. (TIF) [file pone.0266648.s006.tif]

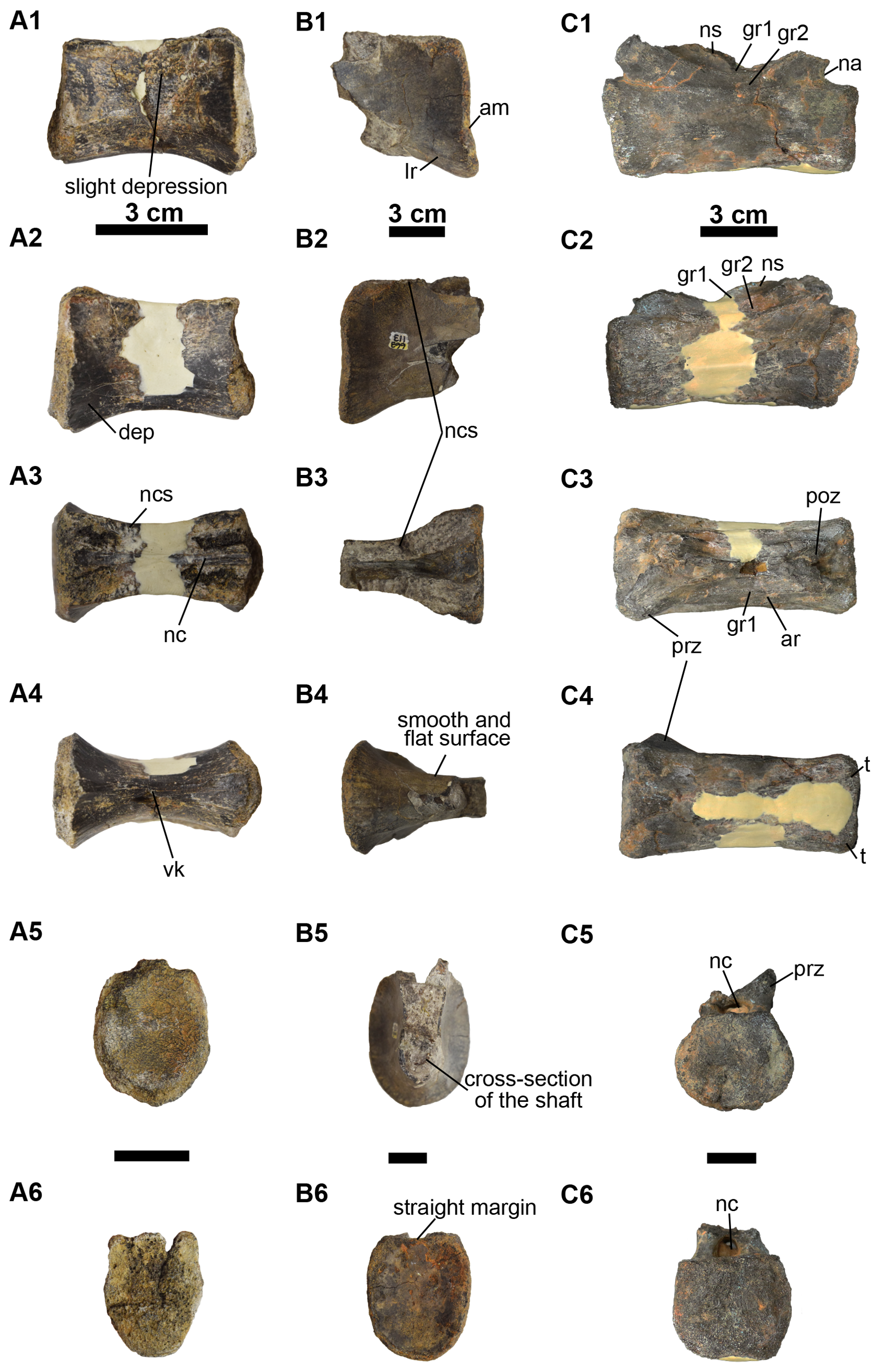

Supplement: S7 Fig — (A), the anterior dorsal (MMNS VP-6120); (B), the posterior dorsal (MMNS VP-113); (C), the posterior caudal centra. (A1-C1), left lateral; (A2-C2), right lateral; (A3-C3), dorsal; (A4-C4), ventral; (A5-C5), anterior; and (A6-C6), posterior views. Abbreviations: am, an angled margin of the articular surface; ar, angular ridge; dep, depression; gr, groove; lr, longitudinal groove; na, neural arch; nc, neural canal; ncs, neurocentral suture; ns, neural spine; poz, postzygapophysis; prz, prezygapophysis; vk, ventral keel. Scale bars equal to 3 cm for A1-A4 – C1-C4 and 2 cm for A5-A6 – C5-C6. (TIF) [file pone.0266648.s007.tif]

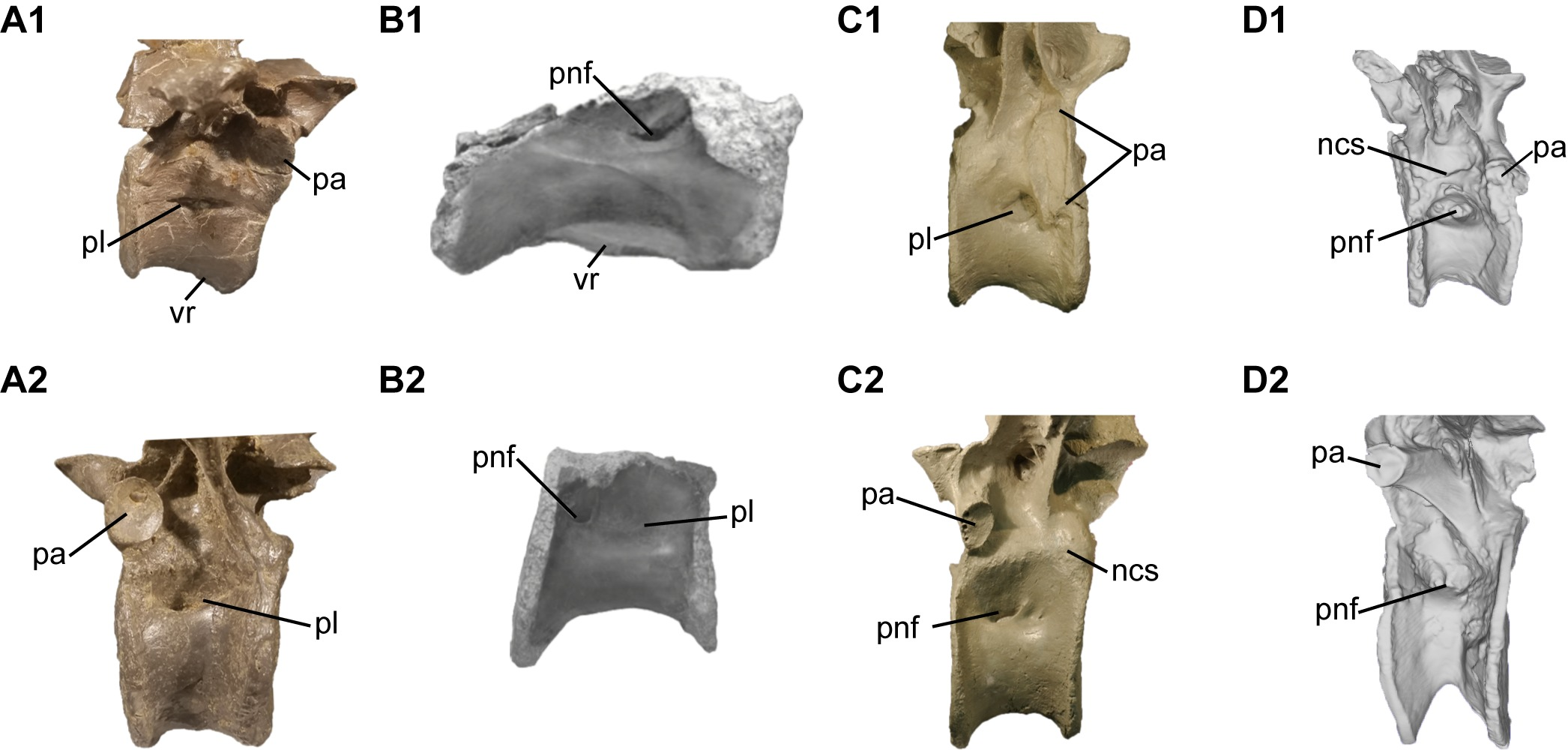

Supplement: S8 Fig — Comparison of the anterior (A) and the posterior (B) dorsal vertebrae of selected theropod dinosaurs. A1, C1-D1, the fourth and A2, C2-D2, the eleventh dorsal vertebrae; (A1-A2), D. antirrhopus (YPM 5204 and 5210) in left lateral views; (B), S. meekerorum in right (B1) and left (B2) lateral views; (C1, C2), A. riocoloradense in left lateral views; (D1, D2), T. rex (FMNH PR 2081, 3D reconstructed image) in right (D1) and left (D2) lateral views. Abbreviations: ncs, neurocentral suture; pa, parapophysis; pl, pleurocoel; pnf, pneumatic fossa. All images are not to scale. (TIF) [file pone.0266648.s008.tif]

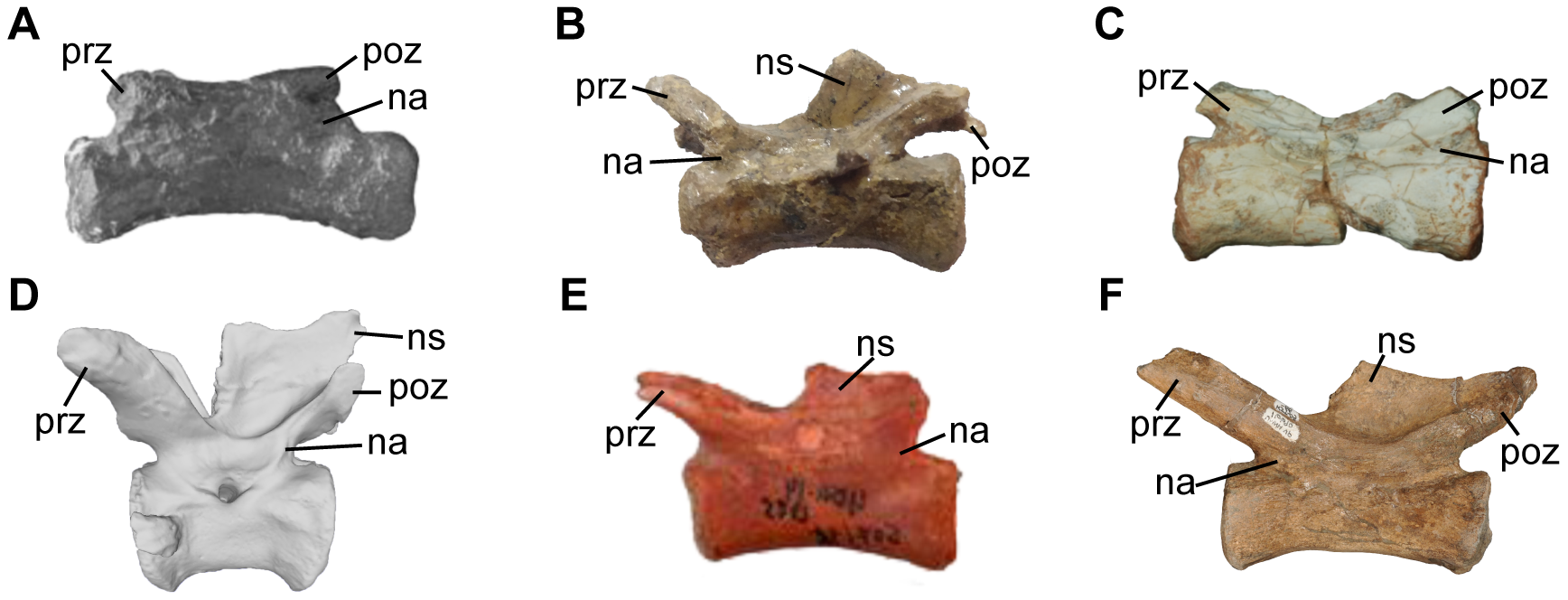

Supplement: S9 Fig — (A), D. aquilunguis (ANSP 9995); (B), M. roseae (MCF-PVPH-247, reversed); (C), Z. salleei; (D), T. rex (FMNH PR 2081, 3D reconstructed image); (E), T. euotica (ZIN PH 507/16); (F), Utah tyrannosaurid (UMNH VP 20200). Abbreviations: na, neural arch; ns, neural spine; poz, postzygapophysis; prz, prezygapophysis. Image modified from (A), Brusatte et al. [100]. All images are not to scale. (TIF) [file pone.0266648.s009.tif]

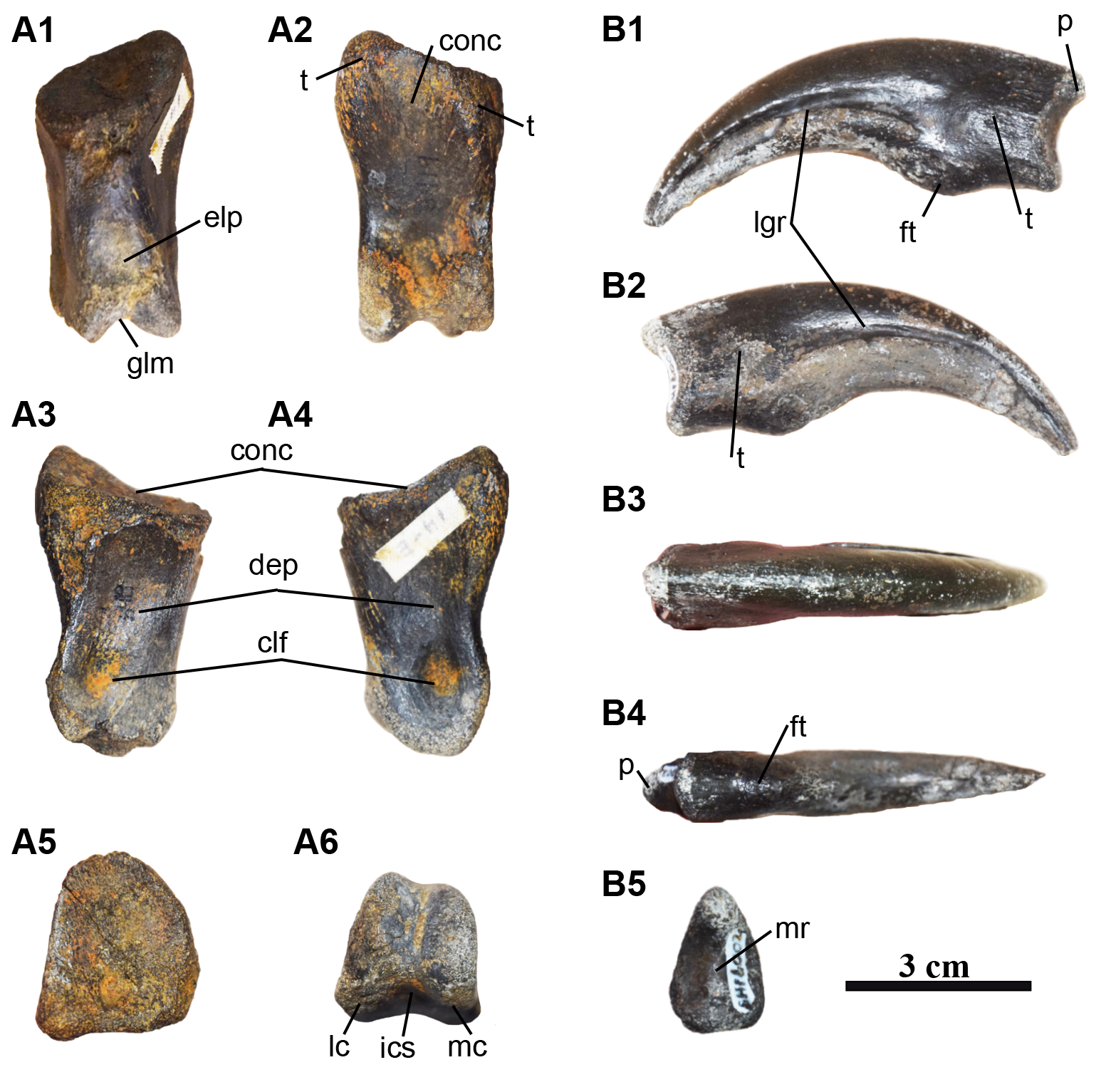

Supplement: S10 Fig — (A), the right first phalanx of digit 3 (PIII-1); (B), the ungual phalanx (I-1?). (A1, B3), anterior; (A2, B4), posterior; (A3, B1), lateral; (A4, B2), medial; (A5, B5), proximal; and (A6), distal views. Abbreviations: clf, collateral ligament fossa; conc, concavity; dep, depression; elp, extensor ligament pit; ft, flexor tubercle; glm, ginglymoid articular surface; ics, intercondylar sulcus; lc, lateral condyle; lgr, lateral groove; mc, medial condyle; mr, medial ridge; p, process; t, tubercle. (TIF) [file pone.0266648.s010.tif]

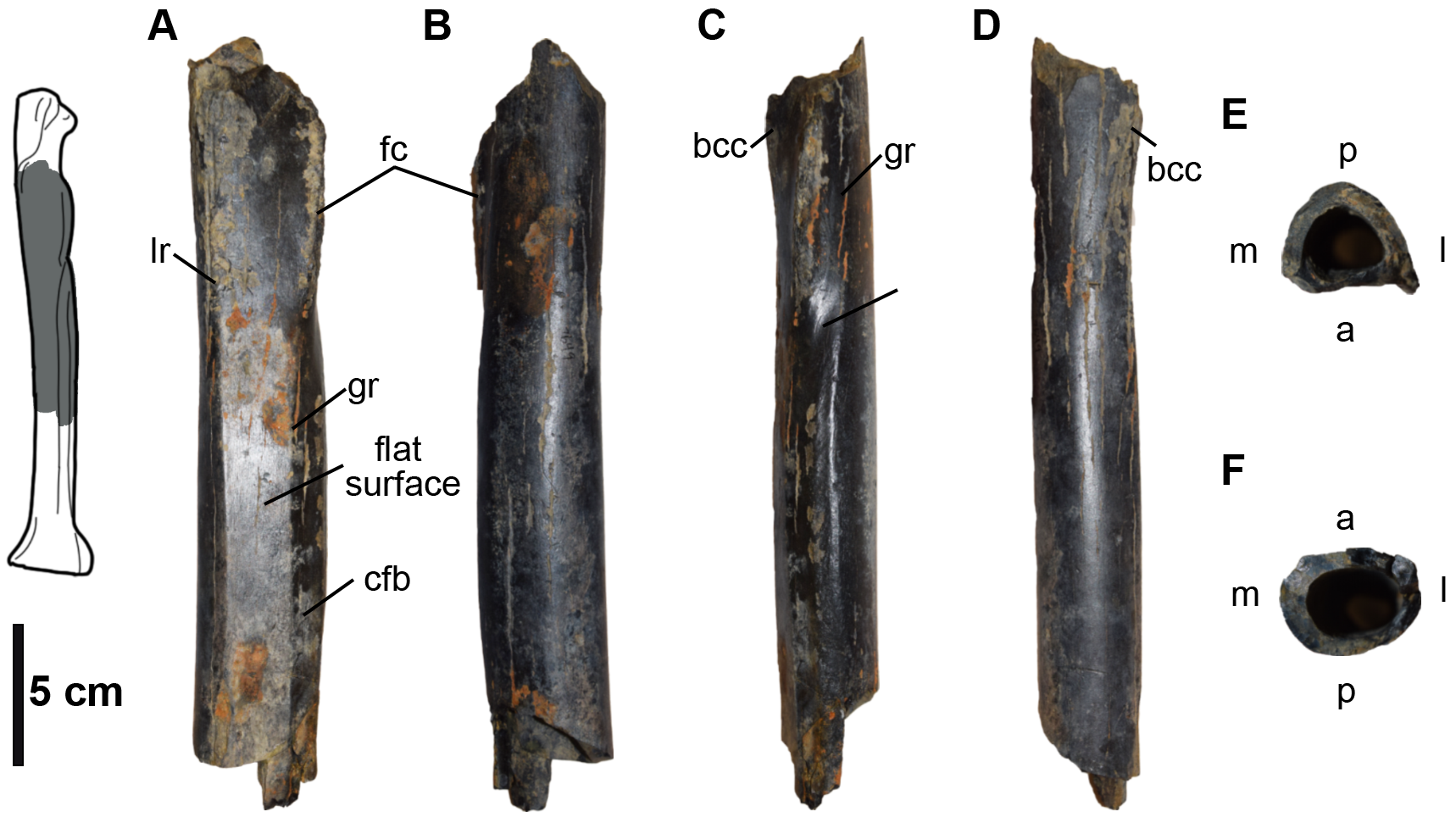

Supplement: S11 Fig — (A), anterior; (B), posterior; (C), lateral; (D), medial; (E), proximal; (F), distal views. Interpretive illustration of Q. henanensis (HGM 41HIII-0106) shows the approximate location of the preserved portion of the midshaft. Abbreviations: a, anterior; bcc, a base of the cnemial crest; cfb, contact surface for the fibula; fc, fibular crest; l, lateral; m, medial; p, posterior. (TIF) [file pone.0266648.s011.tif]

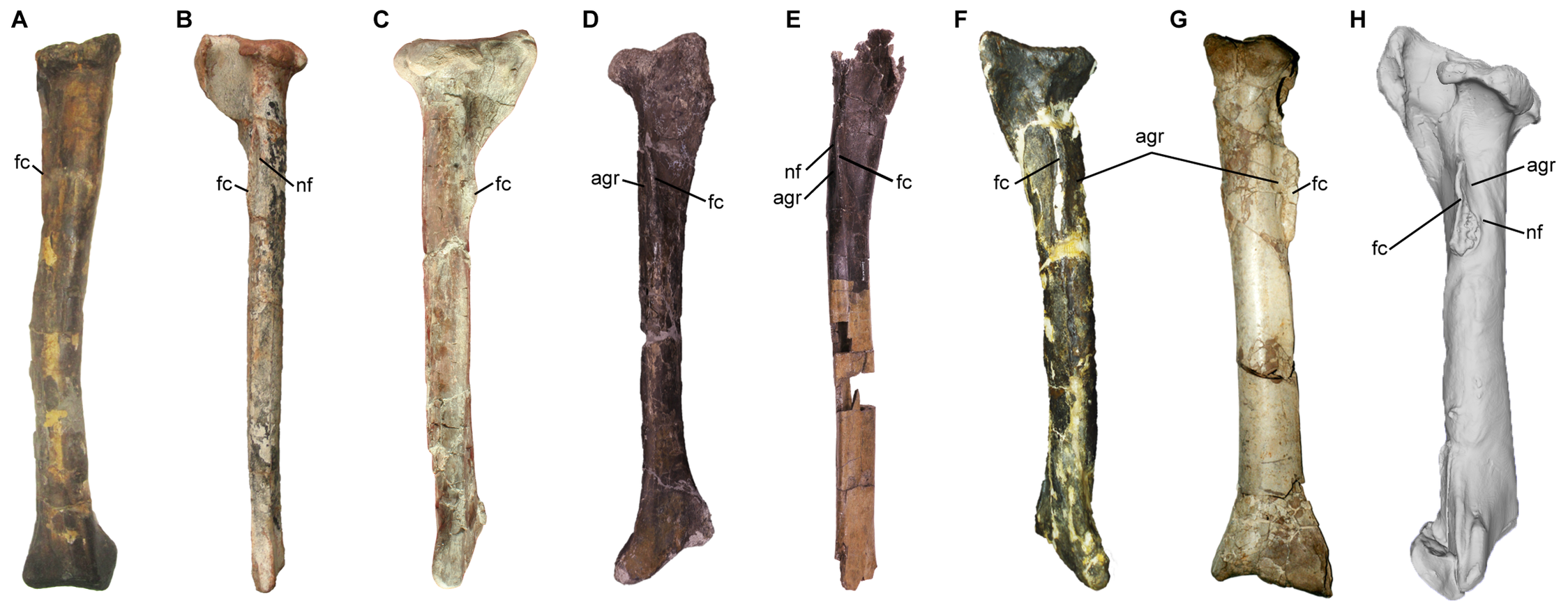

Supplement: S12 Fig — (A), G. bullatus (MPC-D 100/11); (B), Q. henanensis (HGM 41HIII-0106); (C), B. grandis (FRDC-GS GJ 06); (D), Falcarius sp. (CEUM 52393); (E), M. intrepidus (NCSM 33392); (F), D. aquilunguis (ANSP 9995); (G), A. montgomeriensis (RMM 6670); (H), T. rex (FMNH PR 2081, 3D reconstructed image). Abbreviations: agr, a groove of the articular facet for the fibula; fc, fibular crest; nf, nutrient foramen. A, B, D, F, and H are referred to left and C, E, and G are referred to the right tibiae. Note that all images are not to scale and seen from lateral view except for (A) and (G), which are in posterior view. (TIF) [file pone.0266648.s012.tif]
