# Supplementary material for: Large-bodied ornithomimosaurs inhabited Appalachia during the Late Cretaceous of North America
Source: PLoS One. 2022 Oct 19;17(10):e0266648. doi: 10.1371/journal.pone.0266648 (PMC9581415; doi:10.1371/journal.pone.0266648)
Supplement: S1 File — (DOCX) [file pone.0266648.s015.docx]

**Supplementary Material**

**Vertebrae**

The three specimens (MMNS VP-6120, MMNS VP-113, and MMNS VP-6329) from the Mississippi ornithomimosaurs are recovered from the vertebral series (S7 Fig). All of which are mainly composed of the centrum and are missing the upper portions above the neurocentral suture, except the one posterior caudal centrum, which is preserved as a complete neural arch and a part of the prezygapophyses, and the preservation of these centra varies from a near-complete to incomplete. The preserved centra are recognized that they came from different positions of the dorsal and caudal series. Because the centrum is the only available element part of the vertebrae, the characteristics and the investigation that are provided in this study are based on the centrum morphologies for the identification. Furthermore, the present centra appear to belong to different individuals based on their provenance and relative sizes.

**Dorsal vertebrae (MMNS VP-6120 and MMNS VP-113)**

The centra of MMNS VP-6120 and MMNS VP-113 are referred to as the dorsal vertebra (S7A1-A6 and B1-B6 Figs) based on the following features, such as anteroposteriorly elongated, spool-shaped, transversely constricted centrum with a lack of pneumatization, and concave ventral surface, and are represented to Ornithomimosauria. The transversely constricted centrum with a lack of the ventral surface and the absence of chevron facets as well as prominent tubercles on the ventral surface of the centrum exclude these centra from either sacral or caudal vertebrae. Moreover, the different features and sizes of these centra indicate that they belong to different positions of the dorsal series and likely came from different individuals.

The MMNS VP-6120 centrum is herein referred to as one of the anterior dorsal vertebrae by the presence of a well-developed ventral keel (S7A1 Fig). The parts of both anterior and posterior ends and the centrum shaft are missing, and the centrum shaft is reconstructed. The anterior articular surface is less worn than its posterior one, allowing the morphology to be observed. The centrum is anteroposteriorly long relative to its height and the taller than wide centrum has slightly concave, oval-shaped intervertebral articular surfaces (platycoelous), which are perpendicular to the main axis of the centrum in lateral view (S7A1 and A5-A6 Fig). The complete lateral wall of MMNS VP-6120 is smooth and both ends of the centrum deviate outwardly at the intervertebral articular surfaces (S7A3 Fig). The centrum has a slight depression on the dorsolateral surface just below the neurocentral suture. This depression is more visible on the complete right side of the centrum (S7A1 Fig). A similar depression is also present in the anterior dorsal vertebra of the Bissekty taxon [1]. Moreover, none of the parapophysis nor the pleurocoel are evident in the MMNS VP-6120 centrum. The straight neurocentral suture is compressed at the midlength, forming an hourglass shape in the dorsal view (S7A3 Fig). It bears an extremely reduced neural canal throughout its entire length, but the anterior end is slightly widened transversely. Although it is impossible to determine its stage of maturity without the centrum and the neural arch contact at the neurocentral suture, the presence of the visible zigzag pattern of the neurocentral suture, MMNS VP-6120 may belong to an immature individual.

Furthermore, MMNS VP-6120 narrows more ventrally and forms the ventral keel (S7A4 Fig). The ventral keels have been previously reported in the anterior dorsal series of both early-diverging and late-diverging ornithomimosaur taxa, including the fourth dorsal centrum of *Ornithomimus* sp. (TMP 93.62.1) [2], fourth and fifth dorsal centra of *G. brevipes* (Kobayashi and Barsbold 2005*b*, fig. 7), the fifth dorsal centrum of *S. orientalis* [4] and the anterior dorsals of *N. thwazi* [5]*,* and the first, second, as well as the fourth dorsal centra, but the absence in the sixth dorsal centrum of [6]. The ventral keel of MMNS VP-6120 doesn’t occupy the entire ventral surface of the centrum as seen in early-diverging taxa, such as *N. thwazi,* *S. orientalis, H. okladnikovi* and *G. brevipes,* as well as also that of a late-diverging ornithomimid, *Ornithomimus* sp. (TMP 93.62.1). The ventral keel is more pronounced at the anterior two-thirds, but the posterior one-third is flat and forms the anteroposteriorly stretched smooth triangular surface, which is demarcated by a prominent ridge sideways (Fig S7A4)). Whereas the ventral keel tends to be slightly broadened anteriorly, it is suddenly terminated by a flat surface posteriorly in ventral view. Although the anteroventral margin of the articular surface is not complete, the anteroventrally more extended structure in comparison to its posterior end, it may have represented the hypapophysis as in the Bissekty taxon, as well as an early-diverging taxon, *N. thwazi* [5]. Moreover, it has a distinct but shallow anteroposteriorly extended depression on the anteroventral surface of the centrum just above the ventral keel in lateral view (S7A2 Fig). The outline of this depression is roughly subtriangular and is anteriorly broad and deep and is diminished posteriorly. The morphologically similar depression is also observed in the anterior dorsal centrum of *N. thwazi* [5], but, unlike *N. thwazi*, MMNS VP-6120 depression is more distinctive than *N. thwazi.* In addition, a ventral margin of the MMNS VP-6120 ventral keel is sharp, whereas that of *N. thwazi*. has a blunter margin of the ventral keel. The anteroposteriorly elongate, rectangular centrum with a slight ventral excavation in lateral view, a faint groove on the dorsolateral surface, and a sharp ventral keel with a posteriorly flat surface in MMNS VP-6120 resembles those of the fifth dorsal vertebra of *G. brevipes* ([86], fig. 7c, d), and the anterior dorsal vertebra of the Bissekty taxon [1]. However, it differs from these taxa by forming a more anteriorly extended ventral keel and a smooth and flat ventral surface posteriorly. The anteroventrally pronounced ventral keel is also present in some oviraptorosaurs (e.g., *Nomingia gobiensis* [119]) and therizinosauroids (e.g*., F. utahensis* [45]), but the difference in MMNS VP-6120 from these taxa is the sharper ventral keel. Although defining the exact position of MMNS VP-6120 within a vertebral series is difficult based on a single centrum, the absence of the parapophysis and the pleurocoel on the lateral surface suggests that MMNS VP-6120 is not possessed to the anteriormost dorsal vertebra. However, a preferable position would be possibly third or fifth dorsal vertebra, but it was no farther than the sixth dorsal vertebra based on the descriptions of a complete loss of the ventral keel of the sixth dorsal vertebrae in *G. brevipes* and *H. okladnikovi* [3,6]. Furthermore, the straight neurocentral suture, the extended hypapophysis, and the subtriangular articular surface of MMNS VP-6120 is much resembled that of one of the anterior dorsal vertebrae of caenagnathid *E. rarus*, but a dorsally positioned large pleurocoel on the lateral surface of the centrum differentiates it from this taxon [9].

Moreover, the absence of the parapophysis below the neurocentral suture is consistent with ornithomimosaurs, such as *G. bullatus* [10], but it differs from the early-diverging ornithomimosaurs (e.g., *H. okladnikovi*, MPC-D 100/29 [66]), which bears the parapophysis on the lateral surface of the centrum at least up to the fourth dorsal vertebra of the anterior series.

Another partial centrum of MMNS VP-113 was previously briefly described by Carpenter [11] and King and Jones [12] (S7B1-B6 Fig). Whereas Carpenter stated that the MMNS VP-113 centrum belongs to the anterior caudal vertebra of a theropod dinosaur based on the features of the elongate, lateromedial constriction of the central body, King and Jones [12] commented that it belonged to dromaeosaurids without providing any rigorous description. Nevertheless, we disagree with their descriptions, which are inaccurate both morphologically and taxonomically and led to the misidentification of the centrum. If MMNS VP-113 belonged to one of the anterior caudal vertebrae as suggested by Carpenter [11], the centrum would have presented at least one of the following characters in the ventral side. These include a pair of tubercles or the articular facet for the chevron. Although none of these characters is observed in MMNS VP-113, one longitudinally oriented ridge is revealed on the left lateroventral surface of the centrum. Interestingly, this ridge is developed only on one side of the centrum and its general appearance is not consistent with the corresponding pair of tubercles as seen in other theropods (S7B1 Fig). Rather, this irregularly developed ridge represents one type of pathology or not can be determined by CT-image [13]. A lack of these characters is inconsistent with MMNS VP-113 being referable to one of the anterior caudal vertebrae. Furthermore, MMNS VP-113 also doesn’t support the statement of King & Jones [12] by representing the outline of the articular surface and anteroposteriorly elongated centrum without the pleurocoel. Here, we redescribed MMNS VP-113 and assigned it as one of the posterior dorsal vertebrae, referable to Ornithomimosauria based on the following statements [14,15].

MMNS VP-113 is less complete than the MMNS VP-6120 centrum and is composed of only half of the centrum, but is missing all upper parts above the neurocentral suture, similar to the other vertebrae (S7B Fig). Based on the preserved portion, the centrum is anteroposteriorly elongate and it would have been long relative to the dorsoventral height when the centrum is complete (Table 1). The transversely constricted centrum is spool-shaped, and the intervertebral articular surface is taller than wide, sub-oval, and shallowly amphicoelous as in other ornithomimosaurs (S7B3-B4 Fig). Laterally, the intervertebral articular margin is slightly angled at the mid-length (S7B1 Fig). The similarly angled margin is also observed in the posterior articular margin of the posterior dorsal vertebrae of *G. brevipes* [3] and *G. bullatus*. Compared to the posterior margin, the anterior margin of the posterior dorsal vertebra in these taxa is more convex in lateral view. Based on the latter feature as well as taller than wide central body, and the absence of the articular facet for the chevron, MMNS VP-113 represents the posterior half of the posterior dorsal vertebrae. The articular surface of MMNS VP-113 features a straight dorsal margin and convex lateral and ventral margins in posterior view (S7B6 Fig). The relatively straight and less round margin of the MMNS VP-113 intervertebral articular surface is different than those of allosauroids, tyrannosauroids, and therizinosauroids (greater transverse width than the height). Unlike a dorsally excavated articular surface of the dorsal centrum of *T. rex* [13,16,17], MMNS VP-113 has a straight margin in posterior view (S7B6 Fig). However, it is somewhat similar-looking to those seen on the posterior dorsal centra of *A. fragilis* [18]. But the MMNS VP-113 centrum is differentiated from *A. fragilis* by several differentiated features as well as being incomparably small in size. The lateral surface of the centrum shaft of MMNS VP-113 is flat and smooth, and it has U-shaped cross-section as in ornithomimosaurs (S7B4-5 Fig), but it is unlike those in allosauroids, tyrannosauroids, and carcharodontosaurids (e.g., *A. fragilis, T. rex*, and *Siats meekerorum*), which bear a ventrally broadened, cylindrical cross-section of the centrum shaft [13,18–20] (S8B2-D2 Fig). Likewise, in MMNS VP-6120, MMNS VP-113 displays a straight laterally and hourglass dorsally neurocentral suture (S7B2-3 Fig). The neural canal is greatly widened at the anterior and posterior ends relative to the midlength in dorsal view.

Neither the pleurocoel or the distinct depression on the lateral surfaces of the MMNS VP-113 and MMNS VP- 6120 centra is evident, which distinguishes them from those of tyrannosaurids (e.g., *T. rex* [49] and *T. bataar* [74]), as well as gigantic megaraptoran neovenatorids (e.g., *Aerosteon riocoloradense* [124], *S. meekerorum* [123]), and therizinosauroids (e.g., *F. utahensis* [45]) (S8B1-D1 and B2-D2 Fig). Although, in the case of MMNS VP-113, it can be said that the pleurocoel may have been lost with the missing anterior half, it is unlikely based on the presence of anteroposteriorly long, laterally constricted, elongate centrum. Whereas the transversely constricted centrum with a platycoelous articular surface is also present in *S. meekerorum*, the following diagnostic characteristics of *Siats* are different from the dorsal centra of the Eutaw ornithomimosaurs: a dorsally directed, slit-like pneumatic fossa, a ventrally extended apron-like ventral keel of the anterior dorsal vertebrae, and a relatively deep ventral excavation of the posterior dorsal vertebrae [20] (S8B1-2 Fig). Moreover, the absence of a dorsally excavated circular articular surface, and a well-excavated ventral surface of the posterior dorsal vertebra, as in the therizinosauroid *F. utahensis* [8], excludes MMNS VP-113 from therizinosauroids.

Furthermore, both centra belonging to those of the medium-bodied theropod dinosaurs, such as dromaeosaurids (e.g., *D. antirrhopus* [96]) and oviraptorosaurs (e.g., *N. gobiensis* and *H. huangi* [119,125]) is unlikely. Although a general morphology of these dorsal centra, such as a transversely constricted centrum and the presence of the ventral keel, is somewhat similar to that of dromaeosaurids, such as *D. antirrhopus*, several notable characters excluded these centra from this clade [23]. These features include the anteroposteriorly short, box-shaped centrum relative to the height, extremely flared out to the maximum circumference of both ends with a nearly circular articular surface, and a deep, small slit-like pleurocoel just beneath and parallel to the neurocentral suture on the lateral surface of the centrum (S8A1-2 Fig). Thus, the presence of the anteroposteriorly elongate, transversely constricted, ventrally concave, spool-shaped centrum with a lack of the pleurocoel, and dorsoventrally tall, slightly concave (platycoelous) articular surface of these centra most closely resembles Ornithomimosauria.

**Caudal vertebra (MMNS VP-6329)**

An incomplete caudal vertebra of MMNS VP-6329 is composed of the complete centrum and the neural arch but is missing most of the upper parts, including the neural spine, and pre- and postzygapophyses, except the proximal of the left prezygapophysis (S7C1-C6 Fig). The preservation of the MMNS VP-6329 centrum is not fully complete, but it is the most complete centrum, possibly from the Eutaw Formation of the Luxapallila Creek, if any are discovered elsewhere. The anteroposteriorly elongated centrum and the lack of the transverse processes indicate that the MMNS VP-6329 centrum came from the posterior caudal series. The anteroposterior length is 2.5 times longer relative to the dorsoventral height of the anterior articular surface (Table 1), which is greater than the longest caudal centra of any known large-bodied ornithomimosaur taxa. For instance, it is 1.13 times larger than the holotype of *G. bullatus* [10], 1.21 times from the large ornithomimid from the Dinosaur Park Formation [25], and 1.42 times from the holotype of *Paraxenisaurus* [26]. Further, it approaches a size of the posterior caudal vertebra of tyrannosauroids, such as *D. aquilunguis*, which length is ranged between 104 mm and 118 mm [27].

Only the base of the broken neural spine is preserved, which is not informative, so relatively little can be described (S7C1-2 Fig). Although the zygapophyses are broken, the orientation can be determined based on the preserved bases. Whereas the orientation of the prezygapophyses exhibit a wide space between each other as they extend more to anteriorly, the position of the postzygapophyses are parallel to each other along with the posterior end of the centrum in dorsal view (S7C3 Fig). The solely preserved proximal portion of the left prezygapophysis is vertically oriented on the anterior end of the neural arch, which projects anterodorsolaterally in lateral and dorsal views (S7C1, C3 Fig). Based on the preserved portion of left prezygapophysis, the process is beginning to narrow relative to the base, suggesting that the complete prezygapophysis were not long. The neural arch is located slightly anteriorly on the centrum in lateral view, which occupies nearly 80% of the entire length of the centrum (S7C1 Fig). The anteriorly positioned neural arch is different than those of tyrannosauroids, such as *D. aquilunguis* [27]*, T. rex* (FMNH PR 2081), and *G. libratus* (CMN 2120) [13,28], where the neural arch of these taxa are positioned at the center or more posteriorly positioned on the centrum in lateral view (S9A, and E Fig). But, some tyrannosauroids, such as *Timurlengia euotica* and the Utah tyrannosaurid, as well as a tyrannoraptor *Z. salleei* and a carcharodontosaurid *M. roseae* have in a similar manner to MMNS VP-6329 [17,29,30] (S9B-C and E-F Fig). On the lateral surface of the neural arch of MMNS VP-6329, the two distinct grooves are retained on the dorsolateral surface of the centrum. Whereas the thinner and deeper groove is positioned at the base of the neural spine (gr1), the wider and shallower one (gr2) is located ventral to it and is separated by a prominent angular ridge (S7C1-3 Fig). Furthermore, these grooves are slightly bent ventrally at the midlength from the best-preserved left side of the centrum (S7C1 Fig). The neural canal is well-exposed at both ends (S7C5-6 Fig). The anterior neural canal is transversely wider and dorsoventrally flatter than the posterior one. The neurocentral suture is not evident on the MMNS VP-6329 centrum like most theropods.

The anterior and the posterior articular surfaces of MMNS-VP-6329 are different in morphology (S7C5-6 Fig). The anterior end is transversely wider than the posterior one in ventral view and has a teapot-like, slightly concave surface, whereas the posterior articular surface is flat and subquadrangular with the subequal height/width ratio. Morphologically distinct these two articular surfaces in the same centrum are rarely seen in dinosaurs (e.g., [82], fig. 5.12b), but it is possible to make a potential argument here that MMNS VP-6329 may belongs to the transitional vertebra of a tail, where corresponding surfaces of the vertebrae are usually obscured or inaccessible by the adjacent vertebrae in the case of the articulated specimens or it is difficult to ascertain on the isolated vertebrae directly. However, similar features have been reported in carcharodontosaurid *M. roseae* [19] and tyrannoraptor *Z. salleei* [32]. Although MMNS VP-6329 exhibits a similarly displayed distinct outline of the articular surface as in carcharodontosaurid *M. roseae* (fig. 18a-b, 89), the perpendicular intervertebral articular margin relative to the main axis and less ventrally concave MMNS VP-6329 centrum are differentiated it from *M. roseae* (S7C1 Fig).

MMNS VP-6329 is also compared to the posterior caudal vertebrae of the Late Cretaceous Appalachian tyrannosauroids *A. montgomeriensis* and *D. aquilunguis*, which are geographically the closest to MMNS VP-6329 than other known tyrannosauroids [27,33]. The posterior caudal vertebrae of *D. aquilunguis* are generally well-preserved and more complete than one in *A. montgomeriensis*. However, MMNS VP-6329 differs from those of the preserved posterior caudal vertebrae of *A. montgomeriensis* by a transversely less constricted, ventrally straight centrum with perpendicular articular margin to the sagittal plane in lateral view, and prominent lateral grooves (S7C1-3 Fig) [33]. The latter feature is also seen in tyrannosaurids (e.g., *T. rex,* *G. libratus,* and the Utah tyrannosaurid [49,73,126]), as well as carcharodontosaurids, such as *M. roseae* [19].

Recently, the holotype of tyrannosauroid *D. aquilunguis* was redesigned and provided the additional skeletal information, which allows us to make a detailed comparison [27]. In general, the morphology of the MMNS VP-6329 centrum closely resembles *D. aquilunguis* and several similar shared features are present between MMNS VP-6329 and of *D. aquilunguis*, including a wider anterior end than the posterior one ([88], fig. 9d-e), the presence of the angular ridge and longitudinal grooves on the lateral surface of the neural arch ([88], fig. 9b), and the slightly constricted centrum shaft ([88], fig. 9e). However, the following features exclude MMNS VP-6329 from *D. aquilunguis*: (i), a less curved, nearly flat ventral surface in lateral view; (ii), the ventrally bent angular ridge and the grooves in lateral view; and (iii), the presence of the longitudinal ridge on the lateral surface of the centrum. Although the latter feature is also described in the transitional caudal of *G. libratus* [28], and posterior caudals of therizinosauroid *F. utahensis* [8] as well as a noasaurid *V. paranaensis* [34], the position and the number of the longitudinal ridge in these taxa are inconstant between these taxa. For example, whereas the longitudinal ridge is located on the more lateroventral surface in that of *G. libratus* centrum than MMNS VP-6329, *F. utahensis* and *V. paranaensis* exhibit an additional longitudinal ridge along the lateroventral surface of the centrum than MMNS VP-6329 [8,34]. The morphologically similar centra having the longitudinal ridge/transverse process on the lateral surface have been reported in the transitional regions (the anteriormost caudal of the posterior half) of the complete articulated caudal series of the known ornithomimid taxa, such as *S. altus* (AMNH 5339), *D. brevitertius* (CMN 12228), *O. edmontonicus* (TMP 1995.110.1), and *G. bullatus* (ZPAL MgD I/94) [10,35]. Furthermore, Brusatte and colleagues [27] stated that the anteroposteriorly long second angular ridge is only present in the more proximal posterior caudal vertebra and is weak or absent in the succeeding caudals. Based on this statement, they defined the anteriormost posterior caudal vertebra of *D. aquilunguis* from the preserved caudal series. Some features in MMNS VP-6329, such as transversely wider anterior end than the posterior one and dorsoventrally yet flattened articular surfaces, are similar to that of the posterior vertebra of *D. aquilunguis* [27]. The presence of the latter features, MMNS VP-6329 may not be came from far posterior caudal series based on dorsoventrally yet flattened articular surfaces of the centrum. It is also consistent with a statement made by Longrich [25], which was proposed that the features of the dorsoventrally flattened centrum and width of the articular surface are twice as wide as its height are represented to the posterior caudal. With all these features present in MMNS VP-6329, this centrum may be possible to ascertain as the anteriormost centrum of the posterior caudal series. Although the transverse process is completely absent in MMNS VP-6329, it bears a similar angular ridge on the lateral surface close to the neural arch as seen in one of the posterior caudal vertebrae of *D. aquilunguis* ([88], fig. 9b, ANSP 9995). The authors have emphasized that this feature may be an autapomorphic character of *D. aquilunguis* as it is an absent in tyrannosaurids, such as *T. rex* [13]. However, they didn’t formally state this feature as an autapomorphy of *D. aquilunguis* due to the poorly documented nature of the posterior caudal vertebrae of *T. rex* in prior studies. Moreover, two prominent longitudinal grooves are also formed on the upper and lower side of the angular ridge of MMNS VP-6329 in lateral view (S7C1 Fig). The general appearance of these grooves resembles to that of *D. aquilunguis*, which has a thin and deep groove in the upper side, and a wide and shallow groove on the lower side. However, unlike the straight angular ridge and groove of *D. aquilunguis*, the orientation of MMNS VP-6329 has a ventrally bending angular ridge and groove at the mid length of the centrum in lateral view. In contrast to tyrannosauroids, a similarly oriented structure is also frequently seen in other theropods, including ornithomimids (e.g., *G. bullatus* and *S. altus* [14] and tyrannosaurids (e.g., *T. rex* (FMNH PR 2081), [49]). However, the longitudinal groove in tyrannosaurids is different than MMNS VP-6329 by a sharply bending ventrally, forming a “V”-shaped zygapophyseal base in lateral view [13], although some slender footed tyrannosaurids, such as *A. olseni* and *G. libratus* (CMN 2120), exhibit a similar morphology to that seen in MMNS VP-6329.

As noted above, the entire ventral surface of MMNS VP-6329 is largely weathered, which makes it difficult to perform a detailed observation (S7C4 Fig). In general, the ventral surface of MMNS VP-6329 is relatively straight as in ornithomimosaurs in lateral view, when this surface was complete. The degree of the ventral concavity is much less than those in other large theropods, such as allosauroids, tyrannosauroids, and carcharodontosaurids [13,18,19,30], but it is in a similar manner that of *Z. salleei* (S9C Fig). Although the anteroventral rim of the articular surface is damaged, it can be seen that this part was a round outline and no sign of the groove/sulcus as seen in more posterior caudals, which bears a distinct sulcus in the reception of the chevron. In ventral view, a pair of weakly developed tubercles is positioned on the weathered posterior surface of MMNS VP-6329 as in ornithomimosaurs such as *G. bullatus* [10], which also have a well-developed pair of tubercles with a prominent sulcus (S7C4 Fig). Furthermore, the features seen in MMNS VP-6329, such as a low ridge on the sides of the centrum, are also present in most oviraptorosaur dinosaurs. However, several other differential features of oviraptorosaurs are not consistent with MMNS VP-6329, such as the presence of anteroposteriorly short centrum relative to its height with the pleurocoel on the lateral sides of the centrum and a distinct groove on the ventral surface (e.g., *Gobiraptor* [129]). In overall comparisons, MMNS VP-6329 is more closely referrable to Ornithomimosauria based on the following characters: anteroposteriorly elongated, transversely slightly constricted, ventrally less curved, and platycoelous articular facet with a perpendicular intervertebral articular edge in lateral view [14].

**Forelimb elements**

**Manual phalanges**

MMNS VP-6419 and MMNS VP-2963 are represented as the manual phalanges and are referred to as Ornithomimosauria based on the following characters: MMNS VP-2963 is characterized by a slender, mediolaterally constricted, ventrally strongly recurved ungual with a weakly developed distally positioned flexor tubercle, and well-pronounced lateral groove (Makovicky et al. 2004).

**Phalanx III-1 (MMNS VP-6419)**

The MMNS VP-6419 is a relatively complete phalanx, except for the slight missing parts of the outer margin of the proximal end and the articular region of the one side of the distal condyle (S10A Fig). MMNS VP-6419 is referrable to Ornithomimosauria based on the presence of a mediolaterally less constricted straight midshaft with a slightly arched flat ventral surface, and a distally ginglymoid interphalangeal articulation with weakly developed collateral ligament fossae (S10A1-4 Fig). The single concave, taller than wide proximal articular surface, anteroposteriorly taller lateral condyle than the medial condyle with a ginglymoid articular surface, and the expansion of the posteromedial corner of the proximal articular surface indicate that MMNS VP-6419 belongs to a proximal-most phalanx of the right third digit of the forelimb (S10A Fig).

In general, the MMNS VP-6419 phalanx is similar to that of the proximal phalanx of the third digit of ornithomimosaurs, such as *S. altus* (UCMZ VP-1980.1) [37] and *G. bullatus* [10]. The single concave proximal articular surface is consistent with the ball and socket articulation type, which is reported in *S. altus* (UCMZ VP-1980.1) [37]. Nicholls and Russell also stated that this type of the articulation permits considerable flexion, extension, and rotational movement and is found only in ornithomimids. MMNS VP-6419 is proportionately long and proximodistally slender ungual relative to the height of the proximal end, compared to those of the short and blocky proximal phalanges of the third digits of *S. altus* and *G. bullatus* (S10A1-2 and A5 Fig). Because of the proximal phalanges (PhII-1 and PhIII-1) of ornithomimosaur hand are visually very similar each other [38], MMNS VP-6419 could be misidentified due to the isolated condition. However, there are some differences between these two phalanges and the PhIII-1 differs from the PhII-1 by the following characters. For example, the medial margin of the proximal articular surface is flat; the distal condyles of the PhIII-1 are unevenly developed; weakly developed collateral ligament fossae; a shallow intercondylar groove in distal view. The proximal end of MMNS VP-6419 is anteroposteriorly taller than the distal end in lateral view (S10A3-4 Fig). The anterior margin of the proximal articular surface is positioned more distally than the posterior margin in lateral view as in other late-diverging ornithomimosaurs, such as the holotype of *G. bullatus* [10,14], and *S. dongi* [39], but unlike those of the early-diverging taxa, such as *H. okladnikovi* and *D. mirificus*, which the corresponding articular margin is nearly perpendicular to the main axis of the phalanx [6,40]. In lateral view, the margin of proximal articular surface is concave at the midlength, which is different than the straight margin of *G. bullatus* [10] (S10A3-4 Fig). The proximal articular surface is mediolaterally narrowed anteriorly, but it tapers ventrally, which forms a roughly subtriangular in cross-section in proximal view. The outline of the articular surface is straight medially, slightly concave posteriorly, and the convex anteriorly and laterally in proximal view (S10A5 Fig). In lateral view, MMNS VP-6419 is relatively straight anteriorly and slightly arched at the midlength posteriorly in lateral and medial views (S10A3-4 Fig). Unlike most ornithomimosaurs, such as *S. altus* (AMNH 5339, [87]), *G. bullatus* [10], and *S. altus* [39], MMNS VP-6419 bears relatively deep extensor pit just proximal to the distal caput (S10A1 Fig). The orientation of this pit is wider distally and narrower proximally, forming a triangular shape in anterior view. It is delimited by low smooth ridges laterally and medially and is terminated by anterior margin of the distal articular surface in dorsal view. Although *D. mirificus* also bears a similar deep pit in the PhIII-1, the difference of MMNS VP-6419 from the latter taxon exhibits more proximally extended extensor pit [40]. The proximal phalanx (PhIII-1) of the third digit of *S. altus* (UCMZ VP-1980.1) is morphologically the closest resemblance to the MMNS VP-6419 phalanx, which exhibits similarly deep extensor ligament pit and proximodistal elongation of the phalanx relative to its proximal height [37]. The lateral and medial surfaces of MMNS VP-6419 are generally flat and it bears a slight depression proximal to the collateral ligament fossa on each side of the phalanx (S10A3-4 Fig).

The posterior surface of the shaft is generally flat, but it has a shallow concave surface between the tuberosities proximally (S10A2 Fig). This surface is much shallower than in some ornithomimids, such as *S. altus* (e.g., AMNH 5339), and *G. bullatus* (MPC-D 100/11). A pair of tuberosities are weakly developed, but the medial tuberosity is relatively larger and more expanded proximally than the lateral one as like other ornithomimids (Fig. S10A2). In posterior view, MMNS VP-6419 has straight lateral and slightly concave medial margins. This feature is different from those of Asian ornithomimosaurs, such as *D. mirificus, G. bullatus,* and *S. dongi*, where the corresponding margins are relatively straight [37,38]. However, the only taxon that has a similar concave structure is *A. planinychus*. But the position of this structure is opposite in *A. planinychus*, which has a concave lateral margin and straight medial margin in the proximal phalanx of the third digit*.* The lateroventral margin of the phalanx has a relatively sharp ridge than the medial one, which is similar to *O. edmontonicus* (ROM 851), and *S. altus* (e.g., UCMZ VP1980.1).

Distally, the caput of MMNS VP-6419 is slightly rotated clockwise in distal view. The lateral condyle is slightly taller than the medial one, but they are subequal transversely like other theropods (S10A6 Fig). The distal condyles are separated by a deep sulcus, forming a ginglymoid articular surface. It displays a well-developed intercondylar groove between the condyles.

The collateral ligament fossae are weakly developed like most ornithomimosaurs and are located closer to the ventral margin than the dorsal margin of the distal caput like late-diverging ornithomimosaurs, such as *S. altus, O. edmontonicus,* and *G. bullatus* [14] (S10A3-4 Fig). The shape of the fossae is similarly circular, and the medial fossa is slightly larger and deeper than the lateral one.

The MMNS VP-6419 phalanx is also compared to other theropod dinosaurs. Firstly, the features of MMNS VP-6419 are dislike any phalanges of tyrannosaurids, such as *T. rex* (FMNH PR 2081) [13]. However, in some respects, MMNS VP-6419 is similar to those of the first phalanges of the second digit of *G. libratus* and the third digit of *A. fragilis*. In general, they are similar based on features such as a proximodistally elongated phalanx, laterally slightly concave, and the straight medial and ventral and convex dorsal and lateral margins with a single concave proximal articular surface, and the ginglymoid articular surface with a well-developed intercondylar groove. However, the phalangeal midshaft of *A. fragilis* is much more constricted lateromedially and anteroposteriorly, as well as the anteroposteriorly more expanded distal caput with a pronounced collateral ligament fossa in lateral view [18,28]. Moreover, the presence of a less excavated posterior concavity, straight posterolateral margin, and anteroposteriorly taller lateral condyle of MMNS VP-6419 is differentiated it from these taxa.

Furthermore, MMNS VP-6419 is also differentiated from the slender, proximodistally elongated, and more delicate third phalanges of dromaeosaurids (e.g., *D. antirrhopus* [96], *V. mongoliensis* [69,70]), and oviraptorosaurs (e.g., *Apatoraptor pennatus, E. rarus*, and *Hagryphus giganteus* [120,132,133]).

In overall, the presence of taller than wide, single concave proximal articular surface, relatively flat posterior surface with a pair of tuberosities proximally, ginglymoid distal articular surface, and weakly developed collateral ligament fossae of MMNS VP-6419 are more closely characterized as the manual phalanx of Ornithomimosauria.

**Phalanx I-1 (MMNS VP-2963)**

A single, complete ungual phalanx is referrable to the manual ungual phalanx of Ornithomimosauria by the following synapomorphies: elongate, lateromedially constricted, distally positioned weakly-developed flexor tubercle and deep unenclosed grooves each side of the ungual [14,46] (S10B Fig). Manual unguals display variations, which are taxonomically important, in the degree of curvature and elongation, a depth of lateral groove, a shape of articular surface, and a position and size of flexor tubercle [38]. A taller than wide proximal articular surface of MMNS VP-2963 is deeply concave and bears less pronounced process at the anterior articular margin of the proximal end in lateral view (S10B1-2 Fig). The outline of the articular surface is mediolaterally constricted anteriorly and widened posteriorly, forming a tear-drop shaped cross-section in proximal view like other ornithomimosaurs, which exhibits a weakly ginglymoidal articular surface, separating by a weak vertical ridge along the median section as like other ornithomimosaurs [14,25] (S10B5 Fig). The degree of the curvature is strong, which resembles the ungual phalanges of the first digit of early-diverging ornithomimosaurs, such as *N. thwazi* and *H. okladnikovi* and late-diverging ornithomimosaurs, such as *G. bullatus* (MPC-D 100/11), *S. dongi*, and *Struthiomimus* sp. (TMP 90.26.) [14,37,39,46]. However, mediolaterally constricted, slender configuration of MMNS VP-2963 closely resembles the ungual of *S. altus* than those of the stout unguals of the following taxa, *H. okladnikovi*, *G. bullatus*, and *D. mirificus* [38,46]. Moreover, MMNS VP-2963 exhibits the unenclosed lateral grooves in both sides, which are slightly wider proximally than distally where the two grooves are deeper than proximally (S10B1-2 Fig). Distally, the grooves merge at the tip of the ungual in anterior view. On each side of the proximal half of the ungual, it has a faint tubercle, which is located on the proximal to the lateral groove (S10B1-2 Fig). The flexor tubercle of MMNS VP-2963 is weakly developed, bulbous, and is positioned distally from the articular surface (S10B1-2 and B4 Fig). The surface between the articular surface and the flexor tubercle of MMNS VP-2963 is slightly concave in lateral view. The slightly concave surface is similar to the manual unguals of *G. bullatus*, *O. edmontonicus*, *S. dongi*, and *S. altus,* but it differs from those of early-diverging ornithomimosaurs, such as *H. okladnikovi*, *H. qingyi*, and *S. orientalis*, which display a relatively straight surface. The distally positioned flexor tubercle of MMNS VP-2963 is well resembled to the late-diverging taxa, *G. bullatus*, *O. edmontonicus*, *S. dongi*, and *S. altus* but it differentiates from those of *D. mirificus*, *H. qingyi*, and *S. orientalis*, in which exhibit closely positioned flexor tubercles.

The morphology of the ungual is differentiated from those of dromaeosaurids, oviraptorosaurs, and tyrannosaurids. Although the mediolateral compression, strong ventral curvature, and well-exposed concave articular surface of MMNS VP-2963 are similar to dromaeosaurids and oviraptorosaurs, which are also known as *D. aquilunguis* [23], and oviraptorosaurs, such as *A. wyliei* and *H. giganteus* [44,47].

**Hindlimb elements**

**Tibia**

MMNS VP-7649 is an incomplete tibia, comprising a portion of the midshaft, missing both proximal and distal ends (S11 Fig). The presence of a base of the cnemial crest anteromedially and the fibular crest anterolaterally and the absence of the proximal region of the astragalar contact mark distally on the anterior surface of the midshaft indicates that MMNS VP-7649 is referrable to the two-thirds of the proximal shaft of the left tibia (S11A and D Fig). The MMNS VP-7649 shaft is uncrushed, which is preserved its original shape in cross-section (S11E-F Fig). MMNS VP-7649 is slender, proximodistally elongate, hollowed, and mediolaterally slightly wider than anteroposteriorly as like other theropods. Unlike a straight shaft of tibiae of alvarezsaurid *Albertonykus borealis,* and the caenagnathids *A. wyliei* and *E. rarus* [47,48], the proximal half of the MMNS VP-7649 shaft is slightly bowed medially as in ornithomimids (e.g., *G. bullatus*, *S. altus,* and *Q. henanensis*) in both anterior and posterior views (S11A-B Fig). Although tyrannosauroids (e.g., *D. aquilunguis* and *M. intrepidus*), and therizinosauroid (e.g., *F. utahensis*) also have a similarly bowed shaft in their tibia [8,49,50], the degree of MMNS VP-7649 is much lesser than those taxa. Only the distal portion of the cnemial crest is preserved in MMNS VP-7649, which is positioned parallel to the proximal end of the fibular crest on the anteromedial surface along the shaft in anterior view (S11A Fig). Unlike some oviraptorosaurs (e.g., *A. wyliei* and *Avimimus portentosus* [52,134] and slender-footed tyrannosauroids (e.g., *G. libratus* [28], and *M. intrepidus*), a base of the cnemial crest of MMNS VP-7649 is transversely stout like ornithomimosaurs, such as *Q. henanensis,* and *S. dongi* [14,39,51]. A well-pronounced longitudinal ridge runs just distal to the base of the cnemial crest along the anteromedial surface of MMNS VP-7649 (S11A Fig). This ridge is more pronounced as it goes distally, which is consistent with those of ornithomimosaurs, but it is inconsistent with other theropods, such as tyrannosauroids (e.g., *A. montgomeriensis* and *D. aquilunguis*), dromaeosaurids (e.g., *D. antirrhopus*, *V. mongoliensis* [MPC-D 100/986]), oviraptorosaurs (e.g., *A. portentosus*, *Chirostenotes pergracilis*, *E. rarus,* and *G. erlianensis*) and therizinosauroids (*Falcarius* sp.), where this ridge is faintly visible or the absence with a round outline of the shaft in cross-section [52]. Although the similar longitudinal ridge is also present in the following taxa, such as *A. wyliei* (CM 78000) [47] and *Saurornitholestes* (TMP 1988.121.0039), the ridge in these taxa is less distinguishable, as well as they are oriented differently than the one in MMNS VP-7649. For instance, the *A. wyliei* (CM 78000) ridge is bent laterally at the midpoint in anterior view (52, fig.4j). Furthermore, the anterior surface of MMNS VP-7649 is flat and divided by a low groove (S11A Fig). The medial anterior surface is transversely wider than the lateral one and is facing straight anteriorly, whereas the thin one is facing anterolaterally. Two flat surfaces run parallel to each other distally from distal to the fibular crest in the rest of the preserved diaphysis in anterior view. The thin anterolaterally-facing surface is presumably a contact surface for the fibula. The orientation of the fibular contact surface varies in theropods [14]. Whereas the fibular contact surfaces of caenagnathids (e.g., *A. wyliei*, *E. rarus,* and *G. erlianensis*), and dromaeosaurids (e.g., *D. antirrhopus*) often display on the lateral to the posterolateral surface of the tibia [9,23,47,53], a more anteriorly positioned fibular contact surface is typical of ornithomimosaurs. The anterolaterally oriented fibular contact surface of MMNS VP-7649 is consistent with *O. edmontonicus* (TMP 1983.127.0001) and *Q. henanensis* [51,52].

Although tibiae of both MMNS VP-7649 and the holotype of *O. velox* are incomplete, the preserved distal-most shaft of MMNS VP-7649 and the proximal-most shaft of *O. velox* appear to overlap in these taxa and are identical at this section by having anterior and anterolaterally directed flat surfaces [54]. The anterolaterally directed fibular articular surface is terminated distal to the fibular crest and is abruptly insinuated behind the fibular crest from anterior view (S11C Fig), whereas wider anteriorly directed flat surface continues as far as the base of the cnemial crest. The incomplete fibular crest of MMNS VP-7649 is missing its proximal end and the margin. The length of the preserved fibular crest is proximodistally 76.5 mm and extends the anterolateral surface along the midshaft. Although it is incomplete, crescentic, stout morphology of the well-developed fibular crest resembles ornithomimosaurs, such as *A. asiaticus*, *Q. henanensis*, and *D. brevitertius,* and some tyrannosauroid taxa, such as *A. montgomeriensis* and *M. intrepidus* (S12 Fig) [33,49,51,55–57].

Moreover, the morphology of MMNS VP-7649 is also compared to those of other theropod clades, including therizinosauroids (e.g., *F. utahensis*), caenagnathids (e.g.*, A. wyliei*, *C. pergracilis, E. rarus,* and *G. erlianensis* [9,47,53,58], and dromaeosaurids (e.g., *D. antirrhopus* and *Utahraptor* [96]), which are characterized by a nearly flat, straight, and gracile fibular crest (S12 Fig). In addition, oviraptorosaurs differentiate from MMNS VP-7649 by having a distally extending distinct ridge of the fibular crest [7,9]. Furthermore, while the nutrient foramen is occasionally present in some ornithomimosaur taxa, such as *Q. henanensis* and *Ornithomimus* sp. (TMP 1994.012.1010), it is common in tyrannosauroids, caenagnathids and therizinosaurids [13,33]. Unlike representatives of these theropod groups, MMNS VP-7649 does not bear the nutrient foramen on the posterior surface of the fibular crest (S11B-C Fig). Instead, distinctly grooved surface exhibits vertically immediately on the posterior surface of the fibular crest (S11C Fig). Although this grooved surface is also present in other theropods, such as tyrannosauroids and caenagnathids, a much shallow groove of MMNS VP-7649 closely resembles to ornithomimosaurs than these theropods. Just distal to the fibular crest, it exhibits a shallow canal. This canal forms the lateral corner and is further confluent with a longitudinally extended rugose ridge along the lateral surface between the flat anterior and convex posteromedial surfaces like *T. rex* [13].

The outline of cross-section of the North American theropods’ tibiae has previously been suggested as a potential compelling feature for distinguishing theropod superfamilies [52]. Based on the comparisons of the cross-sectional tibial shapes of theropods, the North American ornithomimids are similar to other theropods, such as alvarezsaurs, troodontids, and tyrannosaurs, which exhibit elliptical or circular cross-section in that is defined as a round anterior surface. However, in the case of MMNS VP-7649, it is somewhat different from these theropods that exhibits anteriorly flat surface, which resembles to those of caenagnathids, such as *E. rarus* and UALVP 57349 than ornithomimids (e.g., *O. edmontonicus* [TMP 1983.127.0001]). Although anteriorly flat surface of the MMNS VP-7649 shaft is similar to those of oviraptorosaurs and dromaeosaurids, it is differentiated from these theropod taxa by the following features. Whereas the anteriorly flat and the posteriorly round cross-section of MMNS VP-7649 is distinguished from those of the anteroposteriorly flattened, a somewhat rectangular cross-sectional shape of dromaeosaurid tibiae (e.g., *Saurornitholestes*), the cross-sectional shape of MMNS VP-7649 is distinguished from those of caenagnathid taxa, such as *E. rarus* and UALVP 57349, by exhibiting a well-pronounced flat articular surface for the extensive fibular contact anteriorly (S11E-F Fig). Furthermore, the outline of the cross-section of MMNS VP-7649 is differentiated from those of elliptical or round cross-sectional shapes of other theropod tibiae, such as *T. rex, Troodon formosus,* and *A. borealis* [52]. In addition, the thickness of the shaft wall of MMNS VP-7649 is also different, compared to other theropods. Whereas the thickness of the shaft wall of MMNS VP-7649 is much thinner than those of *T. rex, T. formosus,* and *A. borealis*, it is similar to those caenagnathids (see the detail description in a histological description and Fig 3A). However, MMNS VP-7649 bears relatively thin lateral shaft compared to those of the caenagnathid taxa, such as *E. rarus* and UALVP 57349, which is consistent with that of *O. edmontonicus* [52]. In overall, the presenting features of MMNS VP-7649 are closely similar to ornithomimosaurs (e.g., *O. edmontonicus, O. velox, G. bullatus,* and *Q. henanensis*) than any other theropods.

**References**

1. Sues H-D, Averianov A. Ornithomimidae (Dinosauria: Theropoda) from the Bissekty Formation (Upper Cretaceous: Turonian) of Uzbekistan. Cretac Res. 2016;57: 90–110. doi:10.1016/j.cretres.2015.07.012

2. Makovicky PJ. Phylogenetic aspects of the vertebral morphology of Coelurosauria (Dinosauria: Theropoda). 1995.

3. Kobayashi Y, Barsbold R. Reexamination of a primitive ornithomimosaur, Garudimimus brevipes Barsbold, 1981 (Dinosauria: Theropoda), from the Late Cretaceous of Mongolia. Can J Earth Sci. 2005;42: 1501–1521. doi:10.1139/e05-044

4. Ji Q, Norell MA, Makovicky PJ, Gao K, Ji S, Yuan C. An early ostrich dinosaur and implication for ornithomimosaur phylogeny. Am Museum Novit. 2003;3420: 1–19.

5. Choiniere JN, Forster CA, De Klerk WJ. New information on Nqwebasaurus thwazi, a coelurosaurian theropod from the Early Cretaceous Kirkwood Formation in South Africa. J African Earth Sci. 2012;71–72: 1–17.

6. Kobayashi Y, Barsbold R. Anatomy of Harpymimus okladnikovi Barsbold and Perle 1984 (Dinosauria; Theropoda) of Mongolia. In: Carpenter K, editor. The carnivorous dinosaurs. Indianapolis: Indiana University Press; 2005. pp. 97–126.

7. Barsbold R, Osmόlska H, Watabe M, Currie PJ, Khishigjav. A new oviraptorosaur (Dinosauria, Theropoda) from Mongolia:the first dinosaur with a pygostyle. Acta Palaeontol Pol. 2000;45: 97–106.

8. Zanno LE. Osteology of Falcarius utahensis (Dinosauria: Theropoda): characterizing the anatomy of basal therizinosaurs. Zool J Linn Soc. 2010;158: 196–230. doi:10.1111/j.1096-3642.2009.00464.x

9. Currie P, Funston G, Osmolska H. New specimens of the crested theropod dinosaur Elmisaurus rarus from Mongolia. Acta Palaeontol Pol. 2015. doi:10.4202/app.00130.2014

10. Osmόlska H, Roniewicz E, Barsbold R. a new dinosaur, Gallimimus bullatus n. gen. , n. sp. (Ornithomimidae) from the Upper Cretaceous of Mongolia. Acta Palaeontol Pol. 1972;27: 103–143.

11. Carpenter K. The Oldest Late Cretaceous dinosaurs in North America? Mississippi Geol. 1982;3: 1–7.

12. King TD, Jones ED. Late Cretaceous dinosaurs of the southeastern United States. Gulf Coast Assoc Geol Soc Trans. 1997;47: 263–269.

13. Brochu CA. Osteology of Tyrannosaurus rex : Insights from a nearly complete skeleton and high-resolution computed tomographic analysis of the skull. J Vertebr Paleontol. 2003;22: 1–138. doi:10.1080/02724634.2003.10010947

14. Makovicky PJ, Kobayashi Y, Currie PJ. Ornithomimosauria. 1st ed. In: Weishampel DB, Dodson P, Osmólska H, editors. The Dinosauria. 1st ed. Berkeley: University of California Press [AQ1]; 2004. pp. 137–150. Available: http://dinosauria.ucpress.edu.

15. Buffetaut E, Suteethorn V, Tong H. An early “ostrich dinosaur” (Theropoda: Ornithomimosauria) from the Early Cretaceous Sao Khua Formation of NE Thailand. Geol Soc London Spec Publ. 2009;315: 229–243. doi:10.1144/SP315.16

16. Osborn HF. Tyrannosaurus, Upper Cretaceous carnivorous dinosaur (second communication). Am Museum Nat Hist. 1906;22: 281–296.

17. Averianov A, Sues H-DD, Averianov A, Sues H-DD. Skeletal remains of Tyrannosauroidea (Dinosauria: Theropoda) from the Bissekty Formation (Upper Cretaceous: Turonian) of Uzbekistan. Cretac Res. 2012;34: 284–297. doi:10.1016/j.cretres.2011.11.009

18. Madsen JH. Allosaurus fragilis: a revised osteology. 1993;109: 1–164.

19. Coria RA, Currie PJ. A new carcharodontosaurid (Dinosauria, Theropoda) from the Upper Cretaceous of Argentina. GEODIVERSITAS. 2006;28: 71–118.

20. Zanno LE, Makovicky PJ. Neovenatorid theropods are apex predators in the Late Cretaceous of North America. Nat Commun. 2013;4: 2827. doi:10.1038/ncomms3827

21. Maleev EA. Giant carnivorous dinosaurs of Mongolia. Dokl Acad Sci USSR. 1955;104: 634–637.

22. Sereno PC, Tan L, Brusatte SL, Kriegstein HJ, Zhao X, Cloward K. Tyrannosaurid Skeletal Design First Evolved at Small Body Size. Science (80- ). 2009;326: 418–422. doi:10.1126/science.1177428

23. Ostrom JH. Osteology of Deinonychus antirrhopus, an unusual theropod from the Lower Cretaceous of Montana. Bull Peabody Museum Nat Hist. 1969;30: 1–165.

24. Osmolska H, Currie PJ, Rinchen B. Oviraptorosauria. The Dinosauria. 2004. pp. 165–183.

25. Longrich N. A new, large ornithomimid from the Cretaceous Dinosaur Park Formation of Alberta, Canada: Implications for the study of dissociated dinosaur remains. Palaeontology. 2008;51: 983–997. doi:10.1111/j.1475-4983.2008.00791.x

26. Serrano-Brañas CI, Espinosa-Chávez B, Maccracken SA, Gutiérrez-Blando C, de León-Dávila C, Ventura JF. Paraxenisaurus normalensis, a large deinocheirid ornithomimosaur from the Cerro del Pueblo Formation (Upper Cretaceous), Coahuila, Mexico. J South Am Earth Sci. 2020;101: 102610. doi:10.1016/j.jsames.2020.102610

27. Brusatte SL, Benson RBJ, Norell MA. The Anatomy of Dryptosaurus aquilunguis (Dinosauria: Theropoda) and a Review of Its Tyrannosauroid Affinities. Am Museum Novit. 2011;3717: 1–53.

28. Lambe LM. The Cretaceous Theropodous Dinosaur Gorgosaurus. Geol Surv. 1917;83: 1–85.

29. Zanno LE, Loewen MA, Farke AA, Kim G-S, Claessens LPAM, McGarrity CT. Late Cretaceous theropod dinosaurs of southern Utah. In: Titus AL, Loewen MA, editors. At The Top of the Grand Staircase: The Late Cretaceous of Southern Utah. Indiana University Press, Bloomington, Indiana; 2013. pp. 504–525.

30. Brusatte SL, Averianov A, Sues H-D, Muir A, Butler IB. New tyrannosaur from the mid-Cretaceous of Uzbekistan clarifies evolution of giant body sizes and advanced senses in tyrant dinosaurs. Proc Natl Acad Sci. 2016;113: 3447–3452. doi:10.1073/pnas.1600140113

31. Holtz TR. Tyrannosauroidae. 2nd ed. In: Weishampel DB, Dodson P, Osmόlska H, editors. The Dinosauria. 2nd ed. Berkeley: University of California Press [AQ1]; 2004. pp. 111–136.

32. Choiniere JN, Clark JM, Forster CA, Xu X. A basal coelurosaur (Dinosauria: Theropoda) from the Late Jurassic (Oxfordian) of the Shishugou Formation in Wucaiwan, People’s Republic of China. J Vertebr Paleontol. 2010;30: 1773–1796. doi:10.1080/02724634.2010.520779

33. Carr TD, Williamson TE, Schwimmer DR. A new genus and species of tyrannosauroid from the Late Cretaceous (Middle Campanian) Demopolis Formation of Alabama. J Vertebr Paleontol. 2005;25: 119–143.

34. Langer MC, Martins N de O, Manzig PC, Ferreira G de S, Marsola JC de A, Fortes E, et al. A new desert-dwelling dinosaur (Theropoda, Noasaurinae) from the Cretaceous of south Brazil. Sci Rep. 2019;9: 9379. doi:10.1038/s41598-019-45306-9

35. McFeeters B, Ryan MJ, Schröder-Adams C, Cullen TM. A new ornithomimid theropod from the Dinosaur Park Formation of Alberta, Canada. J Vertebr Paleontol. 2016;4634: e1221415. doi:10.1080/02724634.2016.1221415

36. Lee S, Lee Y-N, Chinsamy A, Lü J, Barsbold R, Tsogtbaatar K. A new baby oviraptorid dinosaur (Dinosauria: Theropoda) from the Upper Cretaceous Nemegt Formation of Mongolia. Hubbe A, editor. PLoS One. 2019;14: e0210867. doi:10.1371/journal.pone.0210867

37. Nicholls EL, Russell AP. Structure and function of the pectoral girdle and forelimb of Struthiomimus altus (THeropoda: Ornithomimidae). Palaeontology. 1985;28: 643–677.

38. Chinzorig T, Kobayashi Y, Tsogtbaatar K, Currie PJ, Takasaki R, Tanaka T, et al. Ornithomimosaurs from the Nemegt Formation of Mongolia: manus morphological variation and diversity. Palaeogeogr Palaeoclimatol Palaeoecol. 2018;494: 91–100. doi:10.1016/j.palaeo.2017.10.031

39. Kobayashi Y, Lü J. A new ornithomimid dinosaur with gregarious habits from the Late Cretaceous of China. Acta Palaeontol Pol. 2003;48: 235–259. Available: http://app.pan.pl/acta48/app48−235.pdf

40. Osmόlska H, Roniewicz E. Deinocheiridae, a new family of theropod dinosaurs. Acta Palaeontol Pol. 1970;21: 5–19.

41. Osborn HF. Skeletal adaptations of Ornitholestes, Struthiomimus, Tyrannosaurus. Am Museum Nat Hist Bull. 1917;35: 733.

42. Norell MA, Makovicky PJ. Important features of the dromaeosaur skeleton: Information from a new specimen. Am Museum Novit. 1997;3215: 1–28.

43. Norell MA, Makovicky PJ. Important features of the dromaeosaur skeleton. II. Information from newly collected specimens of Velociraptor mongoliensis. Am Museum Novitates1. 1999;3282: 1–45.

44. Zanno LE, Sampson SD. A new oviraptorosaur (Theropoda, Maniraptora) from the Late Cretaceous (Campanian) of Utah. J Vertebr Paleontol. 2005;25: 897–904.

45. Funston GF, Currie PJ. A new caenagnathid (Dinosauria: Oviraptorosauria) from the Horseshoe Canyon Formation of Alberta, Canada, and a reevaluation of the relationships of Caenagnathidae. J Vertebr Paleontol. 2016;36. doi:10.1080/02724634.2016.1160910

46. Russell DA. Ostrich dinosaurs from the Late Cretaceous of Western Canada. Can J Earth Sci. 1972;9: 375–402.

47. Lamanna MC, Sues H-D, Schachner ER, Lyson TR. A New Large-Bodied Oviraptorosaurian Theropod Dinosaur from the Latest Cretaceous of Western North America. Evans DC, editor. PLoS One. 2014;9: e92022. doi:10.1371/journal.pone.0092022

48. Funston G, Medonca SE, Currie PJ, Barsbold R. Oviraptorosaur anatomy, diversity and ecology in the Nemegt Basin. Palaeogeogr Palaeoclimatol Palaeoecol. 2018;494: 101–120.

49. Zanno LE, Tucker RT, Canoville A, Avrahami HM, Gates TA, Makovicky PJ. Diminutive fleet-footed tyrannosauroid narrows the 70-million-year gap in the North American fossil record. Commun Biol. 2019;2: 64. doi:10.1038/s42003-019-0308-7

50. Brusatte SL, Choiniere JN, Benson RB, Carr TD, Norell MA. Theropoda dinosaurs from the Late Cretaceous of Eastern North America: anatomy, systematics, biogeography and new information from historic specimens. J Vertebr Paleontol. 2012;32: 70.

51. Xu L, Kobayashi Y, Lü J, Lee Y-N, Liu Y, Tanaka K, et al. A new ornithomimid dinosaur with North American affinities from the Late Cretaceous Quipa Formation in Henan province of China. Cretac Res. 2011;32: 213–222.

52. Funston GF, Currie PJ. A small caenagnathid tibia from the Horseshoe Canyon Formation (Maastrichtian): Implications for growth and lifestyle in oviraptorosaurs. Cretac Res. 2018;92: 220–230. doi:10.1016/j.cretres.2018.08.020

53. Xu X, Tan Q, Wang J, Zhao X, Tan L. A gigantic bird-like dinosaur from the Late Cretaceous of China. Nature. 2007;447: 844–847. doi:10.1038/nature05849

54. Claessens LPAM, Loewen MA. A redescription of Ornithomimus velox Marsh, 1890 (Dinosauria, Theropoda). J Vertebr Paleontol. 2015; e1034593. doi:10.1080/02724634.2015.1034593

55. Macdonald I, Currie PJ. Description of a partial Dromiceiomimus (Dinosauria: Theropoda) skeleton with comments on the validity of the genus. Can J Earth Sci. 2019;56: 129–157. doi:10.1139/cjes-2018-0162

56. Colbert EH, Russell DA. The small Cretaceous dinosaur Dromaeosaurus. Am Museum Novit. 1969;2380: 1–49.

57. Smith D, Galton P. Osteology of Archaeornithomimus asiaticus (Upper Cretaceous, Iren Dabasu Formation, People’s Republic of China). J Vertebr Paleontol. 1990;10: 255–265.

58. Funston G. Caenagnathids of the Dinosaur Park Formation (Campanian) of Alberta, Canada: anatomy, osteohistology, taxonomy, and evolution. Vertebr Anat Morphol Palaeontol. 2020;8: 105–153. doi:10.18435/vamp29362
